# Structural characterization of the ANTAR antiterminator domain bound to RNA

**DOI:** 10.1093/nar/gkac074

**Published:** 2022-02-12

**Authors:** James L Walshe, Rezwan Siddiquee, Karishma Patel, Sandro F Ataide

**Affiliations:** School of Life and Environmental Science, Faculty of Science, University of Sydney, Sydney, NSW 2006, Australia; School of Life and Environmental Science, Faculty of Science, University of Sydney, Sydney, NSW 2006, Australia; School of Life and Environmental Science, Faculty of Science, University of Sydney, Sydney, NSW 2006, Australia; School of Life and Environmental Science, Faculty of Science, University of Sydney, Sydney, NSW 2006, Australia

## Abstract

Regulated transcription termination provides an efficient and responsive means to control gene expression. In bacteria, rho-independent termination occurs through the formation of an intrinsic RNA terminator loop, which disrupts the RNA polymerase elongation complex, resulting in its dissociation from the DNA template. Bacteria have a number of pathways for overriding termination, one of which is the formation of mutually exclusive RNA motifs. ANTAR domains are a class of antiterminator that bind and stabilize dual hexaloop RNA motifs within the nascent RNA chain to prevent terminator loop formation. We have determined the structures of the dimeric ANTAR domain protein EutV, from *Enterococcus faecialis*, in the absence of and in complex with the dual hexaloop RNA target. The structures illustrate conformational changes that occur upon RNA binding and reveal that the molecular interactions between the ANTAR domains and RNA are restricted to a single hexaloop of the motif. An ANTAR domain dimer must contact each hexaloop of the dual hexaloop motif individually to prevent termination in eubacteria. Our findings thereby redefine the minimal ANTAR domain binding motif to a single hexaloop and revise the current model for ANTAR-mediated antitermination. These insights will inform and facilitate the discovery of novel ANTAR domain RNA targets.

## Graphical Abstract

**Graphical Abstract:**
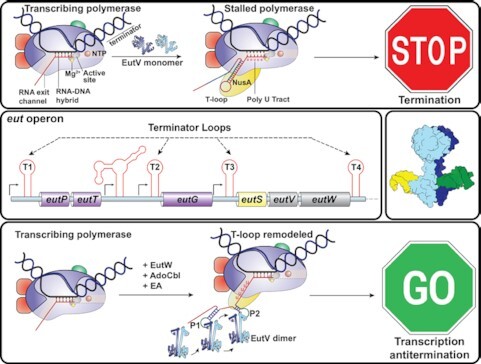
Transcription of the eut operon is regulated by four intrinsic terminator loops. When folded transcription terminates. Activated dimeric EutV promotes formation of an alternative RNA motif enabling transcription to continue.

## INTRODUCTION

**Figure 1.**
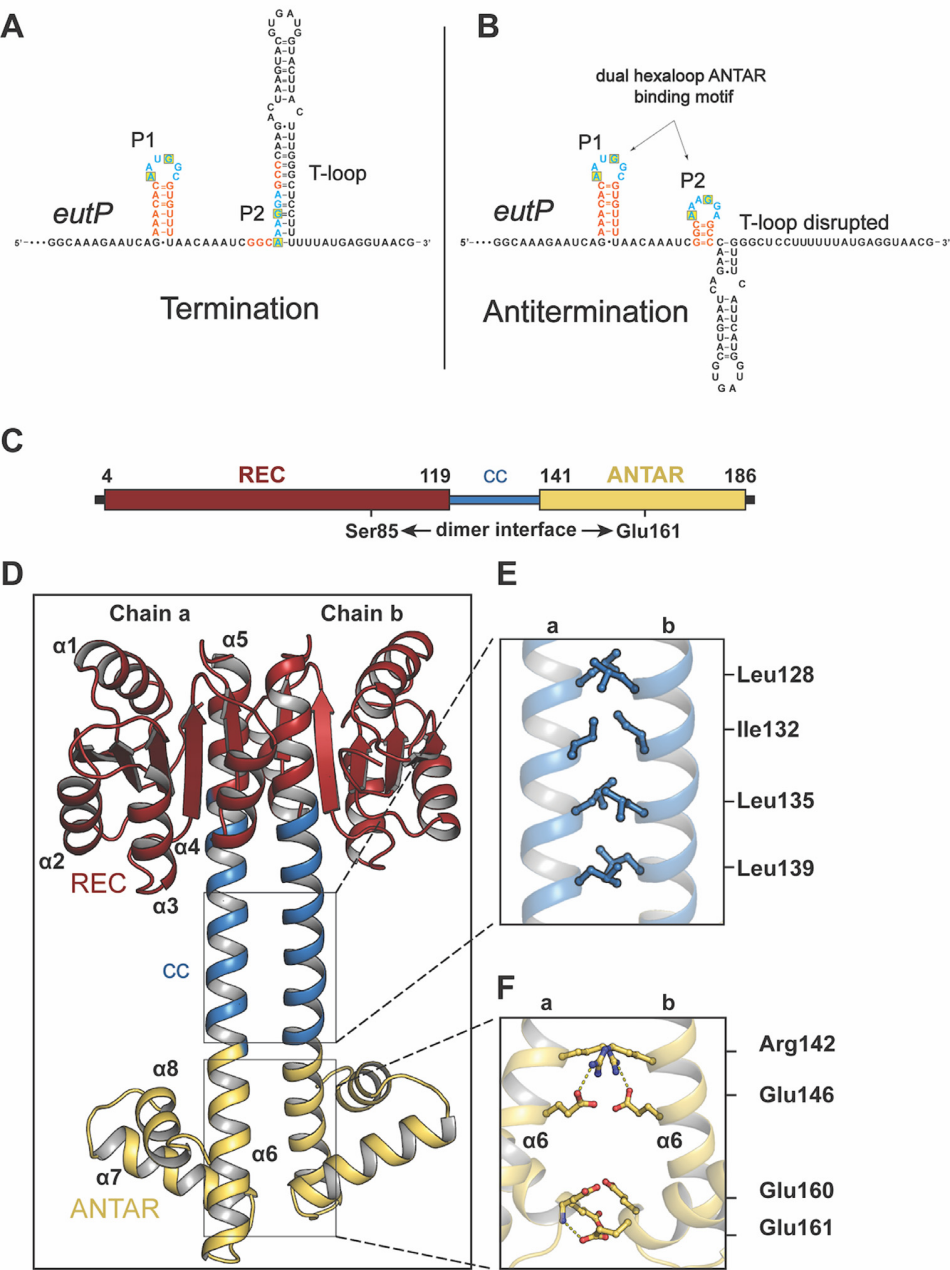
ANTAR binding motif upstream of *eutP* gene and structure of *E. faecalis* antitermination protein EutV. (A, B) Primary sequence of the 5’ UTR of the *eutP* gene from *E. faecalis eut* operon. Bases involved in stem formation are shown in orange, hexaloop bases in blue and conserved bases at position 1 and 4 of the hexaloops highlighted within a yellow box. (**A**) Schematic showing the folding of the intrinsic terminator loop. Only the P1 hexaloop of the ANTAR binding motif is folded with P2 hexaloop embedded within the intrinsic terminator loop. (**B**) Alternative folding for the intrinsic terminator regions to a dual hexaloop motif promoted by the binding of dimeric EutV. Both P1 and P2 hexaloops are folded (26,35,37). (**C**) Schematic representation of dual domain nature of EutV with the receiver (REC) domain and the ANTAR domain shown in red and yellow respectively. (**D**) 2.1 Å resolution crystal structure of EutV captured in a dimeric state highlighting the (**E**) hydrophobic packing of the coiled-coil residues and (**F**) hydrogen bonding between the ANTAR domains.

Transcription can be broadly categorized into three highly regulated processes: initiation, elongation and termination. Termination results in the irreversible dissociation of the RNA polymerase complex (RNAP) from DNA and prevents unintended gene expression, interference from antisense transcripts and provides the cell with a mechanism to rapidly respond to changes in the extracellular environment (1–4). In bacteria, termination occurs either through the action of the Rho RNA helicase or the formation of intrinsic termination loops (T-loops) (1,4). T-loops account for ∼80% of RNA ends in *Escherichia coli* (5) however, their abundance varies across bacterial species (6). Intrinsic termination occurs when the RNAP complex stalls on a poly-uridine tract long enough for the preceding GC rich sequence to fold into a T-loop (Figure 1A) within the RNA exit tunnel thus destabilizing the RNAP complex leading to transcriptional termination (7). T-loops provide a means to demarcate gene boundaries. However, as their formation is passive, bacteria require mechanisms to allow the RNAP to bypass T-loops or prevent T-loops from folding in the first place: a process known as antitermination (2,8)

Passive antitermination mechanisms that affect the formation of T-loops include the action of RNA-binding antiterminator proteins, stalled ribosomes, uncharged tRNAs or small molecules via riboswitches (9–11). Antiterminator proteins may bind specific sequences and/or structural elements in the nascent chain RNA in order to prevent T-loop formation. HutP, from *Bacillus subtilis*, directly binds T-loop sequence repeats to regulate expression of the *hut* operon (12), while the BlgG/SacY/LicT/GlcT family of antiterminators from *B. subtilis* and *E. coli* bind a structured RNA element that precludes the T-loop from forming (13–19). These antiterminator proteins contain vastly different RNA binding domains and display distinct RNA binding mechanisms (Supplementary Figure S1), highlighting the need for individual study of novel antitermination proteins.

The AmiR and NasR transcription antitermination regulator (ANTAR) domain was first characterized as a positive regulator of the *Pseudomonas aeruginosa* amidase operon through AmiR (20–24) and was subsequently identified in the well-studied antiterminator protein NasR (24). ANTAR domains are a novel output domain for two-component signalling (TCS) pathways that, unlike the majority of output domains, bind RNA instead of DNA (25–27). Transduction pathways using TCS exist in bacteria, some archaea, plants and lower eukaryotes (28–30), and allow for the rapid conversion of an external signal to an intracellular response. The ANTAR domain consists of a three-helical bundle of approximately 60 amino acids, with a conserved aromatic residue exposed to the cavity formed by the three-helix structure (Supplementary Figure S2) (24). Proteins containing ANTAR domains are typically modular and include coiled-coil or low complexity domains for dimerization, along with a vast array of sensor or receiving domains such as c**G**MP-specific phosphodiesterases, **a**denylyl cyclases and **F**hlA (GAF) and Per-Arnt-Sim (PAS) domains (Supplementary Figure S2A). The diversity of these accompanying domains is emphasized by the four known structures of proteins containing ANTAR domains (Supplementary Figure S2B–E) (20,31–33) and highlights the large range of bacterial metabolic and regulatory processes that ANTAR domains govern. Current structural studies have focused on the unbound state of ANTAR domains and despite recent efforts to determine residues involved in RNA binding through mutagenesis studies (34), the molecular details of the interactions between an ANTAR domain and its target RNA remains unknown.

Recently, the consensus RNA binding motif for ANTAR domain proteins was characterized in the *Enterococcus faecalis eut* operon, and found to consist of a dual hexaloop motif, with positions 1 and 4 of the hexaloops conserved as adenine and guanine bases respectively (Figure 1B) (26). These motifs are capable of folding within all four T-loops of the *eut* operon (Supplementary Figure S3A and B) with the second hexaloop overlapping with the stem of the T-loop, providing a discernible means for antitermination upon ANTAR domain binding (Figure 1A and B) (26). Furthermore, dual hexaloop motifs have since been identified in other organisms containing characterized ANTAR domain proteins (26,27). Regulation of T-loop formation in the *eut* operon of *E. faecalis*, is under the control of the EutW/EutV TCS pathway. Ethanolamine (EA) stimulates the histidine kinase EutW (component 1) to phosphorylate EutV (component 2) which promotes homodimerization of the protein (Supplementary Figure S3A). Dimeric EutV acts as an antiterminator of RNAP by binding these dual hexaloop motifs within the nascent mRNA chain inhibiting T-loop formation and thereby preventing transcription termination (26,35,36).

ANTAR domains are implicated in the regulation of a wide variety of bacterial processes through both TCS systems or direct coupling to protein sensor domains (23,33,35,37–40) (Supplementary Figure S2A). Additionally, an ANTAR domain involved in the regulation of light sensitive processes, through attachment to a light-oxygen-voltage (LOV) receptor, was recently identified in *Nakamurella multipartita* (Supplementary Figure S2C) (32), paving the way for potential optoribogenetic approaches. Given the expanding interest in ANTAR domains and the plethora of bacterial processes they have been implicated in the regulation of, understanding the molecular details of the interactions between ANTAR domains and their target dual RNA hexaloops is crucial for our understanding of these systems and the potential development of therapeutics to disrupt them. Here we report the first crystal structures of EutV both in the absence of and in complex with the dual hexaloop RNA target. These discoveries allow us to propose a revised model for ANTAR mediated antitermination whereby an ANTAR domain dimer contacts each hexaloop of the dual hexaloop motif successively in order to prevent termination in eubacteria and redefines the minimal ANTAR domain binding motif to a single hexaloop which will facilitate the discovery of novel ANTAR domain RNA targets.

## MATERIALS AND METHODS

### Protein production and purification

The gene sequence for full length EutV/EutW from *E. faecalis* was purchased as a synthetic gene block and cloned into an isopropyl-β-d-thiogalactopyranoside (IPTG)-inducible expression vector containing a TEV protease cleavable his-tag at the N-terminus. For EutV production, *E. coli* Rosetta™ 2(DE3)pLysS cells were transformed with plasmid DNA and were grown overnight on LB agar plates containing 25 μg ml^–1^ Kan and 17 μg ml^–1^ Cam. Large scale cultures, inoculated with resuspended colonies from the transformation plate, were grown at 37°C until an OD_600_ of between 0.3 and 0.4 was reached. The growth temperature was lowered to 18°C and cultures were grown to an OD_600_ of 0.6 when cells were induced with 0.5 mM IPTG and subsequently grown for a further 16–20 h before harvested via by centrifugation. For EutW production, *E. coli* BL21 cells were used.

EutV was purified via a multi-step chromatography protocol consisting of immobilized nickel ion affinity chromatography, proteolytic cleavage of the his-tag, anion exchange chromatography and finally size exclusion chromatography. Briefly, bacterial cells were lysed by sonication in 25 mM sodium phosphate pH 7, 1 M NaCl, 10% (w/v) glycerol, 1 mM TCEP, 5 mM imidazole, 1× cOmplete EDTA-free protease inhibitor, 10 μg ml^–1^ DNase I and 100 μg ml^–1^ lysozyme. The lysate was clarified via centrifugation and the supernatant was applied to a 5 ml HisTrap FF column (Cytiva) to immobilize his-tagged proteins. Bound proteins were washed with buffers containing increasing concentrations of imidazole before final elution with a buffer containing 500 mM imidazole. Proteolytic cleavage of the his-tag using TEV protease was performed in conjunction with overnight dialysis into 25 mM sodium phosphate pH 7, 150 mM NaCl, 10% (w/v) glycerol and 1 mM TCEP in preparation for a second round of immobilized nickel chromatography to remove the cleaved his-tag. To remove contaminating nucleic acids, the sample was then subjected to anion exchange chromatography using a 1 ml ResourceQ column (GE) equilibrated with dialysis buffer, where EutV remained unbound to the column whilst contaminating nucleic acids and proteins were retained. EutV was subsequently concentrated and gel filtration was performed using a Superdex75 column (Cytiva) into a final size exclusion buffer containing 50 mM HEPES pH 7, 300 mM NaCl, 10% (w/v) glycerol and 1 mM TCEP.

The N-terminal EutV-MBP fusion protein was purified in a similar manner to EutV. Clarified lysate was incubated with amylose resin pre-equilibrated with column buffer (20 mM Tris pH 7.4, 200 mM NaCl, 1 mM EDTA, 1 mM DTT). Resin was then washed with 10 CV of column buffer followed by elution with column buffer containing 10 mM maltose. Eluted fractions were combined and subjected to anion exchange chromatography and size exclusion chromatography as described above.

EutW was purified via a multi-step chromatography protocol consisting of immobilized Talon affinity chromatography, anion exchange chromatography and finally size exclusion chromatography. Briefly, bacterial cells were lysed by EmulsiFlex-C3 homogenizer in 25 mM HEPES pH 7.5, 0.5 M NaCl, 2 mM β-ME, 1× cOmplete EDTA-free protease inhibitor, 10 μg ml^–1^ DNase I. The lysate was clarified via centrifugation and the supernatant was applied to 5 ml Talon Superflow beads (Cytiva) to immobilize his-tagged proteins. Bound proteins were washed with buffers containing increasing concentrations of imidazole before final elution with a buffer containing 150 mM imidazole. To further purify the protein, the sample was dialysed to 20 mM Tris–HCl pH 7.5, 75 mM NaCl, 1 mM TCEP then subjected to anion exchange chromatography using a 8 ml MonoQ 10/10 column (Cytiva) equilibrated with dialysis buffer. EutW was bound to the MonoQ column and eluted with a high salt buffer gradient (75–500 mM NaCl) at a flow rate of 4 ml/min. EutW was subsequently concentrated and gel filtration was performed using a Superdex200 column (Cytiva) into a final size exclusion buffer containing 25 mM HEPES pH 7.5, 300 mM NaCl, 1 mM TCEP.

### *In vitro* transcription

For crystallographic studies, a 51-nt RNA (EutP RNA) containing the dual hexaloop motif from the 5’ UTR of the *eutP* gene was produced by *in vitro* transcription. A linearized DNA template containing the EutP RNA sequence, a 5’ T7 RNA polymerase protomer site and a 3′ HDV ribozyme was incubated with T7 RNA polymerase (100 μg ml^–1^, produced in house), pyrophosphatase (10 μg ml^–1^) and RiboSafe RNAse inhibitor (20 U ml^–1^) in transcription buffer (50 mM HEPES pH 7.5, 40 mM MgCl_2_, 100 μg mL^–1^ BSA, 2 mM spermidine, 40 mM DTT and 7.5 mM of each NTP) for 2 h at 37°C before the reaction was finalized at 42°C for 2 h. To ensure maximum folding and subsequent cleavage of the 3′ HDV ribozyme, 50 μg ml^–1^ of an 18-bp oligo (complementary to the 3' end of the RNA) was added post-transcription to prevent secondary RNA structures from inhibiting cleavage. The RNA mixture was then subjected to 2 min heating at 95°C, cooling to 53°C for a further 2 min, followed by rapid cooling on ice for 5 minutes. This process was repeated 2–4 times. The EutP RNA was separated from the template plasmid via denaturing polyacrylamide gel electrophoresis (41) and excised from the gel before extraction by dilution in MQW.

### Electromobility shift assay (EMSA)

*In vitro* transcribed EutP RNA were 5' labelled with Cy5 Maleimide (Kerrafast) and labelling was performed using the 5' EndTag Nucleic Acid Labelling System Kit (Vector Laboratories, Catalog: MB-9001) as per the manufacturer's instructions.

EMSA were carried out using unphosphorylated and phosphorylated EutV and/or EutV-MBP. Varying concentrations of proteins (0–80 μM) were incubated with a fixed 25 nM concentration of Cy5-labelled RNA. Unphosphorylated proteins were mixed with RNA and incubated in binding buffer (25 mM HEPES pH 7.5, 150 mM NaCl, 10 mM MgCl_2_) for 30 min at 4°C. To generate phosphorylated EutV or EutV-MBP, 80 μM of protein, 15 μM EutW, 5 mM ATP, 2.5 mM MgCl_2_, 1 mM Ethanolamine were incubated for 60 min, followed by addition of RNA and another incubation for 30 min at 4°C. The samples were run on 5% (w/v) polyacrylamide gels in 1× TBE buffer at 150 V, 10 mA at a range of durations (60, 45 min) at 4°C.

Mixed gel shift assays were performed by incubating increasing amounts of EutV-MBP (1–30 μM) with a fixed concentration of EutV (80 μM) in binding buffer. The mixtures were phosphorylated by the addition of 15 μM EutW, 5 mM ATP, 2.5 mM MgCl_2_, 1 mM Ethanolamine and incubated for 2 h followed by addition of RNA and a further incubation for 1 h at 4°C. The samples were run on the gel at 150 V, 10 mA, 4°C for 80 min to resolve all possible complexes.

The EutV E140 gel shift assay was performed by mixing the truncated protein at varying concentrations (0–250 μM) with 25 nM RNA in binding buffer and incubated for 30 min at 4°C. The samples were run on the gel at 150 V, 20 mA, 4°C for 20 min. Gels were imaged using a Typhoon FLA 9500 scanner (Cytiva).

### Purifying RNA bound EutV

EutV was incubated with fresh BeF_3_^–^ buffer (30 mM NaF, 5 mM BeSO_4_ and 2.5 mM MgCl_2_) for 1 hour. Precipitation was removed by centrifugation (5 min, 13 500 × *g* and 4°C) before EutV was added to EutP RNA at a three molar excess and incubated for 1 h. Assembled complexes were purified from their individual components by gel filtration in complex-forming buffer (25 mM HEPES pH 7, 150 mM NaCl, 1 mM TCEP, 2.5 mM MgCl_2_ and 2.5% w/v glycerol).

### Size exclusion chromatography (SEC) multi-angle laser light scattering (MALS)

Experiments were performed on an ÄKTA FPLC system (Cytiva) equipped with miniDAWN TREOS MALS and Optilab T-rEX detectors (Wyatt Technology). Purified proteins were separated using either a Superdex75 10/300 or a Superdex200 10/300 Increase (Cytiva) in EutV size exclusion buffer with UV absorbance monitored at 215, 260 and 280 nm. Astra software (Wyatt Technology) was used for data analysis, including baseline and peak broadening corrections using a dn/dc value of 0.1852 ml g^–1^.

### Nuclear magnetic resonance (NMR) spectroscopy

Protein samples were prepared in size exclusion buffer supplemented with D_2_O (10% (v/v)) and 2,2-dimethyl-2-silapentane-5-sulfonic acid (DSS, 150–300 μM). Samples were placed in 3 mm NMR tubes (Shigemi) and spectra were acquired using Bruker Avance III 600 or 800 MHz NMR spectrometers, each fitted with a cryogenic TCI probe-head, at 4°C. Spectra were processed using TOPSPIN (Bruker, Karlsruhe, Germany) and ^1^H chemical shifts were directly referenced to DSS at 0 ppm.

### Far-UV circular dichroism (CD) spectroscopy

Purified protein was dialysed (overnight at 4°C) into 5 mM HEPES pH 7, 300 mM NaF and 1 mM TCEP using 10 kDa molecular weight cut-off dialysis tubing (Progen, Darra, QLD) and diluted to 5–15 μM. CD spectra were recorded using a Jasco J-185 spectrometer (ATA Scientific) at 4°C using a 1 mm quartz cuvette (Sigma-Aldrich). Data were recorded using a speed of 20 nm min^–1^, a response time of 1 s, and a sensitivity of 20 mdeg over the wavelength range 250–195 nm. Data are the average of three scans and were buffer baseline-corrected.

### Surface plasmon resonance

SPR measurements were made using a Biacore T200 instrument (Cytiva). Experiments were performed at 4°C using a multicycle kinetic titration method. 3′ biotinylated RNA constructs (linked via an extended TEG spacer arm) consisting of either the EutP RNA or individual P1 or P2 hairpins were purchased from Integrated DNA technologies (Baulkham Hills, NSW). For comparison of affinities between EutV wildtype and mutants, RNA were immobilized on a Biotin CAPture chip (Cytiva) in 10 mM sodium acetate pH 4.8, 150 mM NaCl, 2.5 mM MgCl_2_ and 0.05% Tween, with a target density of ∼200–250 RU, as per manufacturer's protocol. Different concentrations of proteins were flowed over the reference and RNA-immobilized cells at a flow rate of 50 μl min^–1^ using 10 mM HEPES, pH 7, 150 mM NaCl, 0.05% Tween-20 and 2.5 mM MgCl_2_ as the running buffer. Stripping and regeneration of the chip surface was performed as per the manufacturer's instructions using the supplied reagents. For comparison of affinities between wildtype EutV and phosphorylated EutV, biotinylated RNA was immobilized on a CM5 chip (Cytiva) that had been preactivated with NHS/EDC and loaded with streptavidin, with a target density of 100–200 RU. For phosphorylated EutV, 15 μM of EutW, 5 mM ATP, 2.5 mM MgCl_2_, 1 mM Ethanolamine were incubated for 30 min at room temperature prior to each run. All data were analysed and fit to a 1:1 Langmuir binding isotherm using the Biacore T200 Evaluation Software.

### Statistical analysis

For the SPR experiments, the affinity of EutV to each of the three RNAs (EutP, P1 and P2) were compared using an ANOVA, with the individual means being compared using a Tukey's HSD test to maintain an overall 5% error rate. The affinity of EutV and phosphorylated EutV to EutP RNA were compared with an independent sample *t*-test.

For the *in vitro* antitermination assay, termination for each condition was compared using an ANOVA, with the individual means being compared using a LSD test.

### X-ray crystallography

EutV crystals used for diffraction studies were crystallized in 0.075 M Tris pH 8.5, 18.75% (v/v) *tert*-butanol and 25% (v/v) glycerol using a sitting-drop vapour-diffusion method at 18ºC, with crystals taking between 4 and 10 days to form. The EutV/EutP RNA complex was crystallized in 0.1 M NaCl, 0.1 M HEPES pH 7.5 and 1.4–1.8 M ammonium sulphate with crystals taking 14 days to reach full growth. Crystals were cryoprotected using 25% glycerol and frozen by plunge-freezing in liquid nitrogen. X-ray diffraction data were collected from frozen crystals at the Australian Synchrotron using the Macromolecular Crystallography MX2 beamline (microfocus) at 100 K and a wavelength of 0.9537 Å (42). XDS was used to integrate data and the data were processed further using the CCP4i suite (43,44). Indexing, scaling, and merging of the data was performed using AIMLESS (45,46). Initial phases were calculated by molecular replacement using PHASER (47). The REC domain of Rv1626 (31) was modelled as a dimer using AmiR (20) (PDB: 1S8N and 1QO0 respectively) and used as an initial search model. The models were visualized in COOT (48) and were built manually by iterative rounds of refinement using phenix.REFINE (47) and ISOLDE (49) until convergence. MOLPROBITY (50) was used for structure validation and identification of steric clashes and geometric problems in the final model. Surfaces were evaluated using the web based PISA software (51). The quality of the final models were validated using the wwPDB server and submitted to the PDB (6WSH and 6WW6 for EutV alone and RNA bound respectively). Structure diagrams were generated using PyMOL. The data collection and refinement statistics for these structures are outlined in Supplementary Table S1.

### *In vitro* transcription antitermination assay

The double-stranded DNA templates for *in vitro* transcription antitermination assays consisted of the T7A1 promoter followed by either the EutP RNA or EutP RNA extended linker sequences. Templates finished 53 base pairs after the end of the T-loop. All DNA sequences were purchased from GenScript Biotech and amplified by PCR. Synchronized transcription assays were carried out in 20 μl volumes as described in (52). Template DNA (10 nM) was incubated in 1x Transcription Buffer (20 mM Tris pH 8, 20 mM NaCl, 14 mM MgCl_2_, 14 mM β-ME, 0.1 mM EDTA, 5% glycerol), 12 nM of σ70 saturated *E. coli* RNA polymerase (New England BioLabs), 20 μg/ml of acetylated BSA, 150 μM of the dinucleotide ApU (Tri Link Biotechnologies). 2.5 μM GTP/ATP, 1 μM CTP, 0.33 μM [α-^32^P] CTP (3000 Ci/mmol) was added along with either 1 or 10 μM of EutV wildtype or 1 μM of phosphorylated EutV. The phosphorylated EutV was generated by incubating 40 μM EutV with 15 μM EutW, 1 mM Ethanolamine, 5 mM ATP and 2.5 mM MgCl_2_ prior to addition to the transcription reaction. Components were mixed and incubated at 37ºC for 10 min to generated open stalled complexes at the first U at +30. Synchronized transcription was re-initiated with the addition of 1.5 μl of 10× NTP mix (10 mM ATP, UTP, CTP, GTP and 10 mg/mL Heparin in 1× Transcription Buffer) prewarmed to 37ºC. Reactions were terminated by the addition of an equal volume of 8 M Urea, 1× TBE and 0.05% bromophenol blue [w/v], 0.05% xylene cyanol FF [w/v], resolved via 6% denaturing polyacrylamide gel electrophoresis, visualized using a FLA9500 phosphorimager and quantified using ImageJ software (53).

## RESULTS

### Structure of RNA-free EutV antitermination protein

EutV possesses one of the most common domains architectures found among all ANTAR proteins annotated in the Pfam database (54), consisting of an N-terminal phosphor-sensitive REC domain (aa 1–119) coupled to a C-terminal ANTAR domain (aa 141–186) via a coiled-coil domain (Figure 1C, Supplementary Figure S2). To ascertain the structural arrangements of the ANTAR and REC domains of EutV relative to one another, crystals of recombinantly produced full length EutV were formed, and diffraction data collected to 2.1 Å resolution (Supplementary Table S1, PDB ID: 6WSH). Within the crystal, EutV showed a symmetric dimer ‘dumbbell’ conformation with 48 residues contributing to 1870 A^2^ of buried surface area and extensive hydrophobic and ionic interactions between residues Ser85 and Glu161 of each chain (Figure 1C and D). The REC domain of each chain dimerized through a common ‘α4-β5-α5’ mode (55) forming one end of the dumbbell with the ANTAR domains forming the other end. The handle of the dumbbell consists of a coiled-coil that includes residues Leu128, Ile132, Leu135 and Leu139 and forms the majority of hydrophobic interactions within a heptad repeat (Figure 1E). The interaction between the two ANTAR domains is stabilized by mutual hydrogen bonds between Arg142 to Glu146 (2.9 and 3.0 Å), and the amine group of chain a Glu161 to Glu160 chain b (3.1 Å) (Figure 1F).

The dimeric state within the crystal structure was unexpected given the recombinant EutV used for crystallography was not phosphorylated nor supplemented with phosphomimetics. Analytical size exclusion chromatography (SEC) suggested EutV to be dimeric in solution (Supplementary Figure S4A and B). However previous studies using SEC coupled multi-angled light scattering (MALS) have shown EutV to be monomeric in the absence of phosphorylation (26). SEC-MALS analysis performed in this work indicated EutV was largely monomeric in solution although may form higher order species, likely a dimer at increased concentrations (Supplementary Figure S4C and D). Phosphorylation of REC domains are known to shift the equilibrium of dynamically sampled states, rather than acting as a definitive switch (56–58). In this way, during crystallization and with an increased concentration of EutV, the dimeric state was likely preferentially favoured.

### Structure of RNA bound EutV antiterminator protein

#### Model building the RNA bound EutV structure

**Figure 2.**
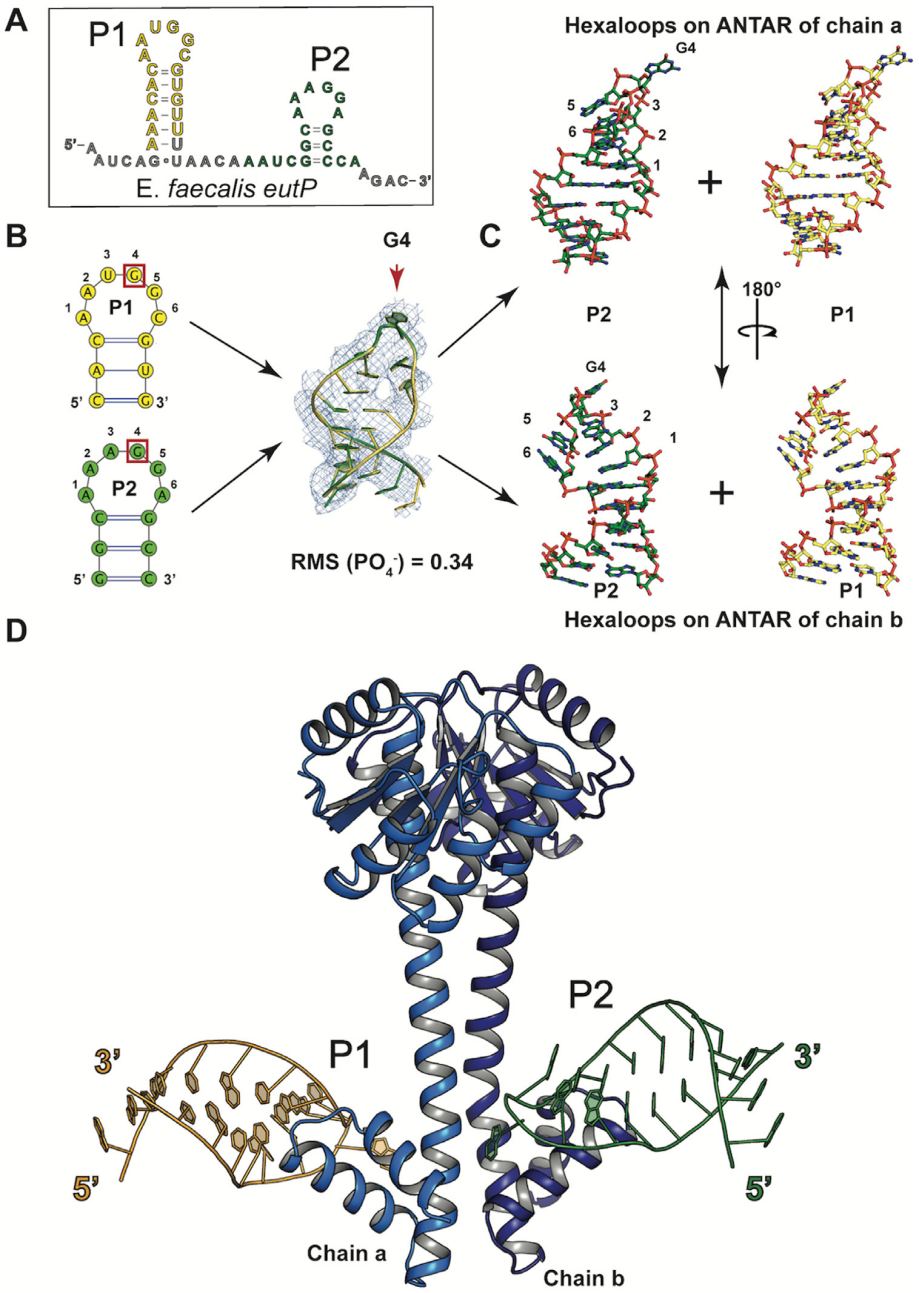
Structure of EutV bound to the dual hexaloop antitermination RNA motif (EutP RNA). (**A**) EutP RNA sequence from *E. faecalis eut* operon within the crystal. P1 and P2 hairpins shown in yellow and green respectively. (**B**) Cartoon representation showing an alignment of the ribose and phosphate backbone of both the P1 and P2 hexaloop from each ANTAR domain of the EutV dimer highlighting the near identical position of the bases. 2*F*_o_– *F*_c_ map contoured to at 1 σ (**C**) Stick representation of each for hexaloops described in (B). (**D**) Crystal structure of EutV bound to EutP RNA motif.

To determine the molecular interactions of the ANTAR domain of EutV with the dual hexaloop RNA substrate, EutV in the presence of the phosphomimetic beryllium fluoride (BeF_3_^–^), was crystallized in complex with a 51-nt EutP RNA (Figure 2A), that corresponds to the 5’ UTR of *eutP* gene, and contains both the P1 and P2 hexaloops (26), to 3.8 Å resolution (Supplementary Table S1, PDB ID: 6WW6, Figure 2). Molecular replacement was performed using the dimeric RNA-free EutV model to estimate initial phases and allowed for the modelling of two asymmetric EutV chains. Additional density present at Asp 54 on both chains (Supplementary Figure S5) indicated successful incorporation of BeF_3_^–^ and activation of EutV. Unambiguous electron density for a single RNA hexaloop was present at the ANTAR domain of each EutV chain (Supplementary Figure S6). Modelling of the entire EutP RNA substrate, with a single hexaloop contacting each ANTAR domain, was not possible given the relative orientation of the hairpins bound at each ANTAR domain. The 5’ ends of each hexaloop were over 70 Å apart with only seven RNA bases unmodeled (Supplementary Figure S7). It became apparent that each of the modelled single hexaloops within the asymmetric unit (ASU) were from different RNA molecules. The dual hexaloop RNA molecule was bridging between two asymmetric units within the crystal lattice with each hexaloop contacting a symmetry-related ANTAR domain of EutV in the neighbouring ASUs (Supplementary Figure S8A and B). To confirm this, the web based RNAComposer software program (59) was used to generate an idealized 3D structure of the EutP RNA. These coordinates, when manually fitted to the positive electron density, were the ideal length to stretch between ANTAR domains from neighbouring ASU in both directions within the crystal lattice (Supplementary Figure S8C and D).

Because each RNA hexaloop (P1 or P2) has a different primary sequence (Figure 2A and B), and each hairpin of the same RNA molecule contacts the symmetry related ANTAR domain in neighbouring ASUs (Supplementary Figure S8D), the electron density for the RNA hairpin at either ANTAR domain within a single ASU represents a combination of the sequences of the P1 and P2 hexaloops (Figure 2B). Within the crystal, whenever a P1 hairpin makes contact with an ANTAR domain within a discrete ASU, the P2 hairpin of the same RNA molecule must contact the symmetry-related ANTAR domain in a neighbouring ASU. Therefore, a model was built by placing both the P1 and P2 hairpins into the density at each ANTAR domain of the dimer, and refinement then performed with a fixed 50% occupancy for all nucleotides (Supplementary Table S1). Despite consisting of different sequences, the P1 and P2 hairpins contacting the ANTAR domains from separate RNA molecules refined to near identical conformations (RMSD = 0.34 Å for the phosphate and ribose backbone) (Figure 2B, Supplementary Figure S9). In total, each ASU contains an asymmetric dimer of EutV and two RNA hexaloops from different RNA molecules (Figure 2D).

**Figure 3.**
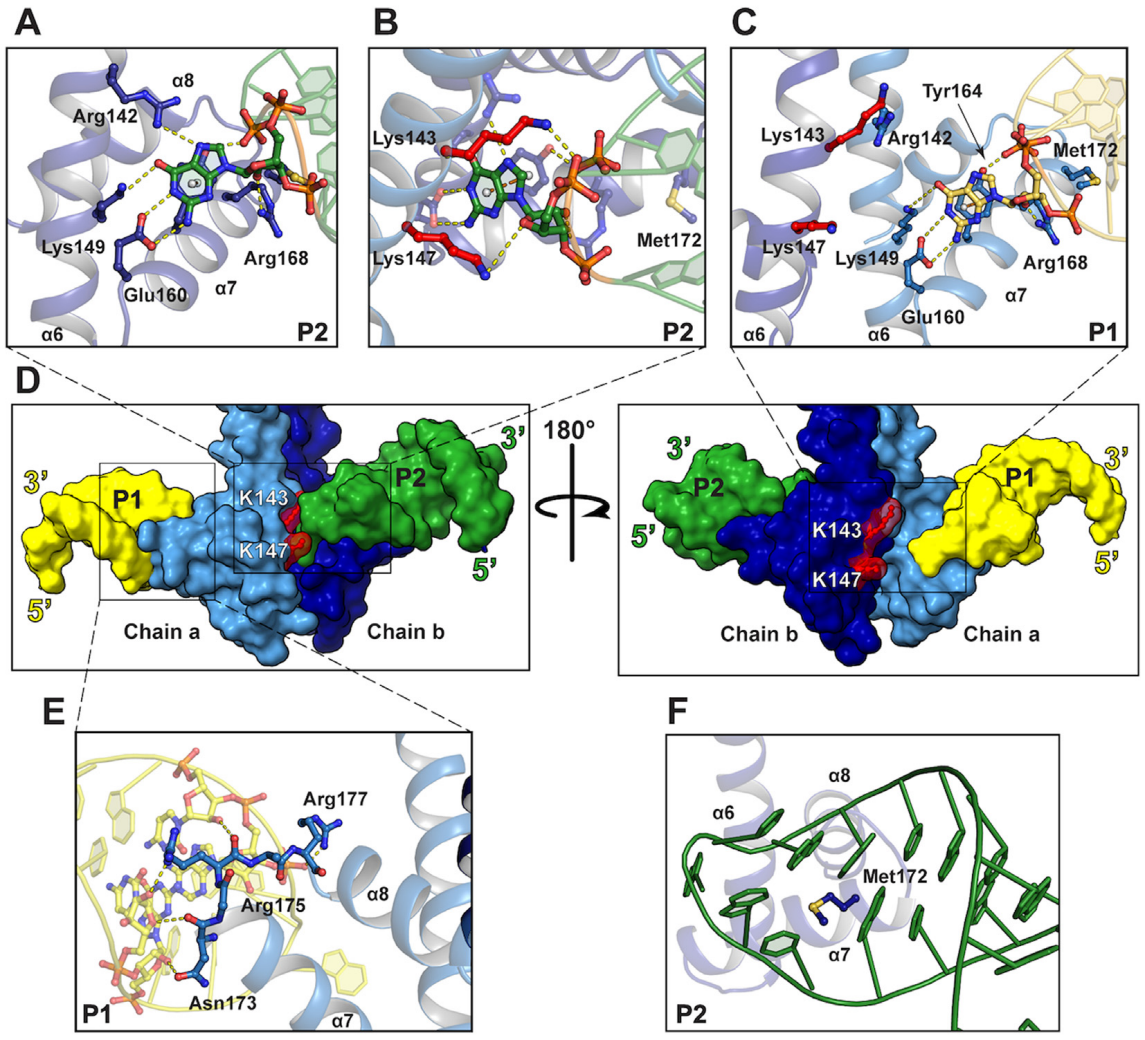
Interactions between ANTAR domain and hexaloop. (**A**) Interactions between the ANTAR domain of chain b and the P2 hexaloop. (**B**) Interaction between chain a ANTAR with the P2 hexaloop. (**C**) Interactions between chain a ANTAR domain and P1 RNA hexaloop. (**D**) Surface representation of the RNA bound EutV structure with Lys143 and L147 highlighted in red (**E**) Interaction between loop α7–α8 loop and P1 hexaloop (**F**) Met172 of chain b ANTAR domain positions P2. Chain a and b shown in light and dark blue respectively. Hydrogen bonds shown as yellow dashes.

In both hairpins, a single base of the hexaloop flips outward to interact with EutV. The flipped nucleotide is either in position 3 or 4 of the hexaloop, depending on the direction the hairpin is modelled (Figure 2B and C, Supplementary Figure S9). The antitermination motif includes a conserved guanosine at positions 4 (G4) of each hexaloop that are obligatory for efficient EutV mediated antitermination *in vivo* (26). Therefore, the flipped base was defined as G4 and used to orientate the direction of the RNA hexaloops on each ANTAR domain. Given this contact is the only base specific interaction between the protein and RNA hairpins (Figure 3, Supplementary Figure S10), and this base is in an identical location in both modelled hairpins (Figure 2B, Supplementary Figure S9), it can be described with confidence despite the averaging effect applied to the electron density. For clarity, only one of the two possible RNA hexaloops at each ANTAR domain will be described in the rest of the manuscript and are labelled P1 and P2 (Figure 2D).

#### Protein:RNA interactions and binding sites

The crystal packing arrangement resulted in a dual occupancy of P1 and P2 hairpins at each ANTAR domain in the ASU therefore applying an averaging affect to the electron density (Figure 2B and C, Supplementary Figure S6). Despite this, successful modelling of the EutV residues that interact with the RNA hairpins was achieved through use of the *ISOLDE* package designed for building high-quality macromolecular models into low to medium resolution experimental maps (Supplementary Figure S11) (49). The interaction between dimeric EutV and the antitermination RNA hexaloops is restricted to the ANTAR domain of each chain, consistent with published gel retardation assays performed using truncated EutV constructs (Figure 2D, Supplementary Figures S7B, S10 and S11) (26). Two putative RNA binding sites with asymmetric protein:RNA interactions were identified on each ANTAR domain of the EutV dimer (Figure 3, Supplementary Figure S10). The different binding modes are due to the large difference between the kink in the coiled-coil region of chain a and that of chain b, which is clearly noticeable when each chain is compared to the RNA-free EutV dimer (Supplementary Figure S12A and B). As a result, chain a and b make more interactions with the P2 RNA hexaloop than P1 (Supplementary Figure S10). Both sites bind a single hexaloop with the conserved G4 of each loop flipping outward to form π–π stacking interactions with Tyr164 of the ANTAR domain (Figure 3A–C, Supplementary Figure S10). Additionally, the hydroxy group of Tyr164 makes a hydrogen bond to the phosphate group of the base in position 3 of the hexaloop (Figure 3C, Supplementary Figure S10). The G4 of P2 is coordinated by hydrogen bonds to Arg142, Lys149 and Glu160 of chain b, however, Arg142 from chain a is unable to make the same contact with P1 (Figure 3, compare A and C, Supplementary Figure S10). Intriguingly, residues Lys143 and Lys147 from chain a contact the P2 RNA, through hydrogen bonds to the phosphate and ribose respectively, representing the only interactions between an ANTAR domain and the RNA hexaloop located on the opposite protein chain (Figure 3B, Supplementary Figure S10A). The prominent nature of the kink in the coiled-coil of chain b prevents the reciprocal interactions between residues Lys143 and Lys147 of chain b existing with P1 (Figure 3D, Supplementary Figure S10B).

On both chain a and chain b, Arg168 is well positioned to hydrogen bond to the ribose hydroxyl group of the RNA (Figure 3A–C, Supplementary Figure S10). Additionally, the residues of the α7–α8 loop make similar contacts with the RNA backbone on either chain. Asn173 and Arg175 hydrogen bond with hydroxyl groups of the ribose sugars of the bases that form the stem of the hexaloop, and Arg177 makes a hydrogen bond to the phosphate backbone of the base at position 2 of the hexaloop (Figure 3E, Supplementary Figure S10). As the interactions are limited to the backbone of the closing three base pairs of the RNA stem, they provide a molecular understanding for the obligatory nature of these stems in *in vivo* antitermination, independent of their sequence (26). Unexpectedly, given its hydrophobic nature, Met172 is positioned in the middle of the hexaloop of the RNA hairpin and may act as a hydrophobic plug to position the hexaloop on α7 within the ANTAR domain and flip the G4 base out of the hexaloop (Figure 3F, Supplementary Figure S10). Met172 may also form potential S–H/π interactions with bases within the hexaloop (60).

### Alanine mutagenesis

**Figure 4.**
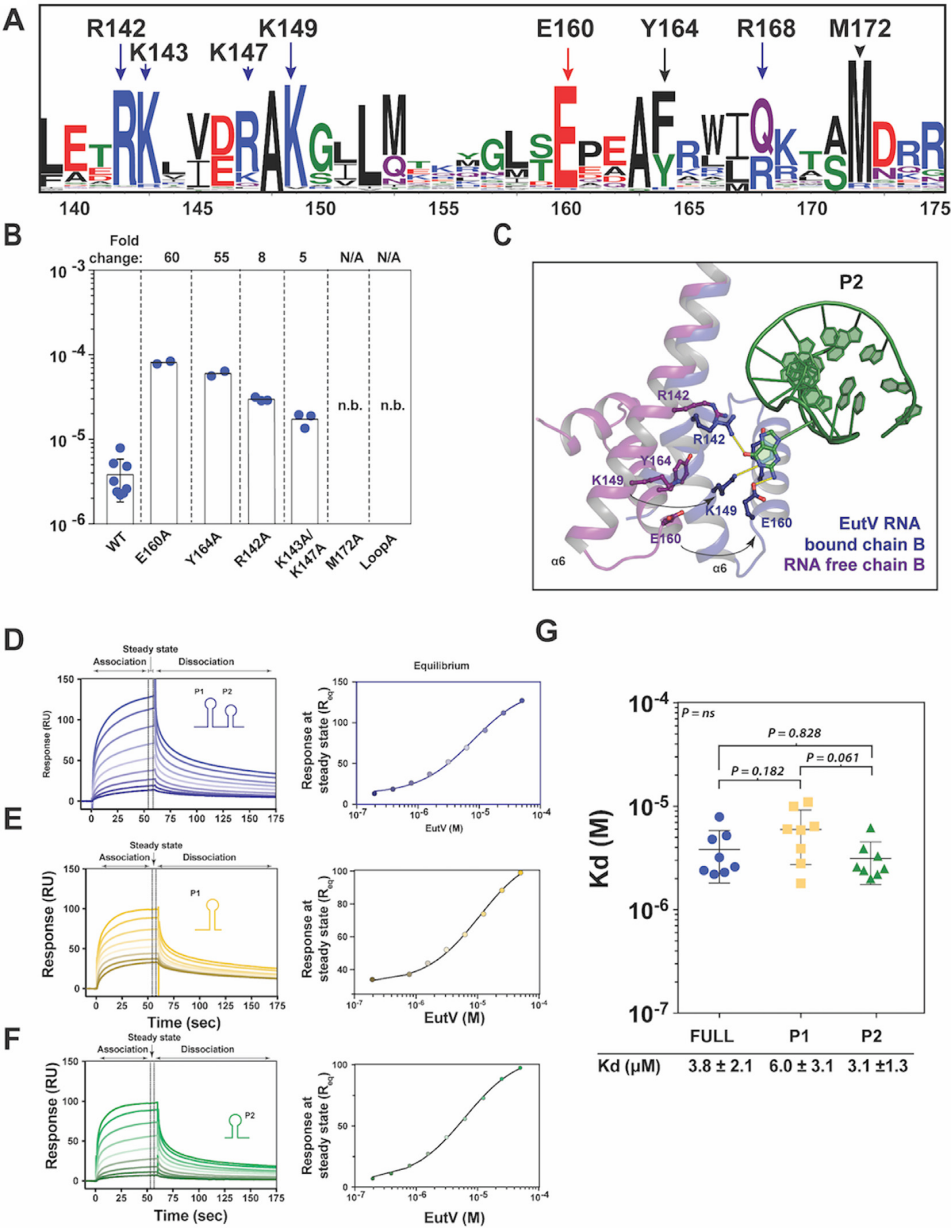
EutV binding to single RNA hexaloops. (**A**) Multiple sequence alignment of 2332 ANTAR domain proteins containing a N-terminal REC domain and C-terminal ANTAR domain shown as a WebLogo (71). (**B**) Binding affinity of EutV mutants to EutP RNA as determined by surface plasmon resonance. (**C**) Movements of residues involved in RNA binding between RNA-free and RNA bound EutV structures. (**D–F**) Representative normalized SPR sensorgrams of EutV binding to the EutP RNA and the P1 and P2 hexaloops. Association and dissociation regions are shown above the panel and apply for all sensorgrams. Sensorgrams showing increasing EutV concentrations (0, 0.4, 0.8, 1.5, 3.125, 6.25, 12.5, 25, 50, 100 μM). Representative dose response plot of the interaction of EutV with immobilized RNA (as shown in corresponding left panel) at equilibrium fitted to a one-site Langmuir isotherm. (**G**) Average experimental *K*_D_ values from eight SPR experiments are 3.8 ± 2.1, 6.0 ± 3.1 and 3.1 ± 1.3 μM for, EutP RNA, P1 and P2 hexaloop respectively. Error bars are standard deviations of eight separate runs. The affinity of EutV to each of the three RNAs was compared using an analysis of variance (ANOVA), with the individual means being compared using a Tukey's HSD test to maintain an overall 5% error rate. The overall *P* value was 0.06 and none of the means were different at the 5% level.

The majority of ANTAR residues implicated in RNA binding are highly conserved (Figures 3A–E, and 4A) (34). To validate the role of these residues, six EutV constructs containing alanine mutations were generated. The folded-states of the mutant constructs were confirmed to be identical to wildtype by circular dichroism and one-dimensional NMR (Supplementary Figure S13A and B), and their RNA binding ability was assessed using surface plasmon resonance (SPR) (Figure 4B and Supplementary Figure S14). Single alanine mutations to the residues involved in base specific interactions with the flipped G4 base resulted in a respective 60- and 55-fold reduction in binding affinity for E160A and Y164A (Figure 3B, Supplementary Figure S14A–C). The third G4 coordinating mutant, R142A, showed a less dramatic decrease in binding with only an 8-fold decrease and the double mutant, K143A/K147A, had a similar modest effect on RNA binding (Figure 4B, Supplementary Figure S14D-E). Drastically, the N173A/R175A/R177A triple mutation and the M172A mutation completely abolished binding to the RNA (Figure 4B, Supplementary Figure S14F–G). The decrease in binding seen across all mutant constructs highlights the significance of the RNA interacting residues identified within the crystal structure and provides a molecular rationale for the findings of the recent mutagenesis studies performed on the ANTAR domain protein NasR from *Klebsiella oxytoca* (34).

### EutV binding to P1 and P2 RNA

The lattice packing within the crystal of EutV bound to RNA suggests that dimeric EutV may bind EutP RNA with a 2:1 stoichiometry, with one dimer binding at each hexaloop (P1 and P2) (Supplementary Figure S8D). To confirm this possibility in solution, electromobility shift assays (EMSA) were performed using the 5’ Cy5 labelled EutP RNA and both EutV or MBP-tagged EutV (EutV-MBP). Both constructs revealed a distinctive double-shift, although this was more prominent with EutV-MBP, confirming two binding sites are present on the EutP RNA. The first shift occurred with EutV concentrations between 5 and 20 μM while the second shift with EutV concentrations between 20 and 40 μM (Supplementary Figure S15A). Phosphorylation of EutV (EutV-P), using the paired histidine kinase EutW, resulted in only a marginal increase in binding to EutP RNA on EMSA (Supplementary Figure S15B). Similarly, a slight increase in EutV-P affinity to EutP RNA (3.5-fold) was seen when measured via SPR (Supplementary Figure S16).

To further confirm a 2:1 binding stoichiometry we carried out additional EMSA experiments using a combination of EutV and EutV-MBP. Various concentrations of the two constructs were mixed, phosphorylated, and subjected to EMSA to delineate binding of either a EutV dimer, EutV-MBP dimer or a mixed dimer containing one subunit of EutV and EutV-MBP (Supplementary Figure S17A). Indeed, mixing EutV and EutV-MBP together constantly produced a shifted band which migrated in between that of a single EutV–MBP dimer and two EutV-MBP dimers binding (Supplementary Figure S17B—compare lane 10, with 11 and 12) strongly suggesting a mixed dimeric complex had formed.

To elicit if EutV displayed a binding preference for either the P1 or P2 hairpin, the binding affinity of EutV to the individual P1 and P2 hairpins were again determined using SPR (Figure 4D–F). No significant difference (*P* = 0.061) was observed between EutV binding to the EutP, P1 and P2 RNAs (6.0 ± 3.1 and 3.1 ± 1.3 μM respectively) (Figure 4G). This was consistent with the crystal packing arrangement where the P1 or P2 hexaloops contact both EutV chains throughout the crystal (Supplementary Figure S8D).

### *In vitro* transcription antitermination assay

**Figure 5.**
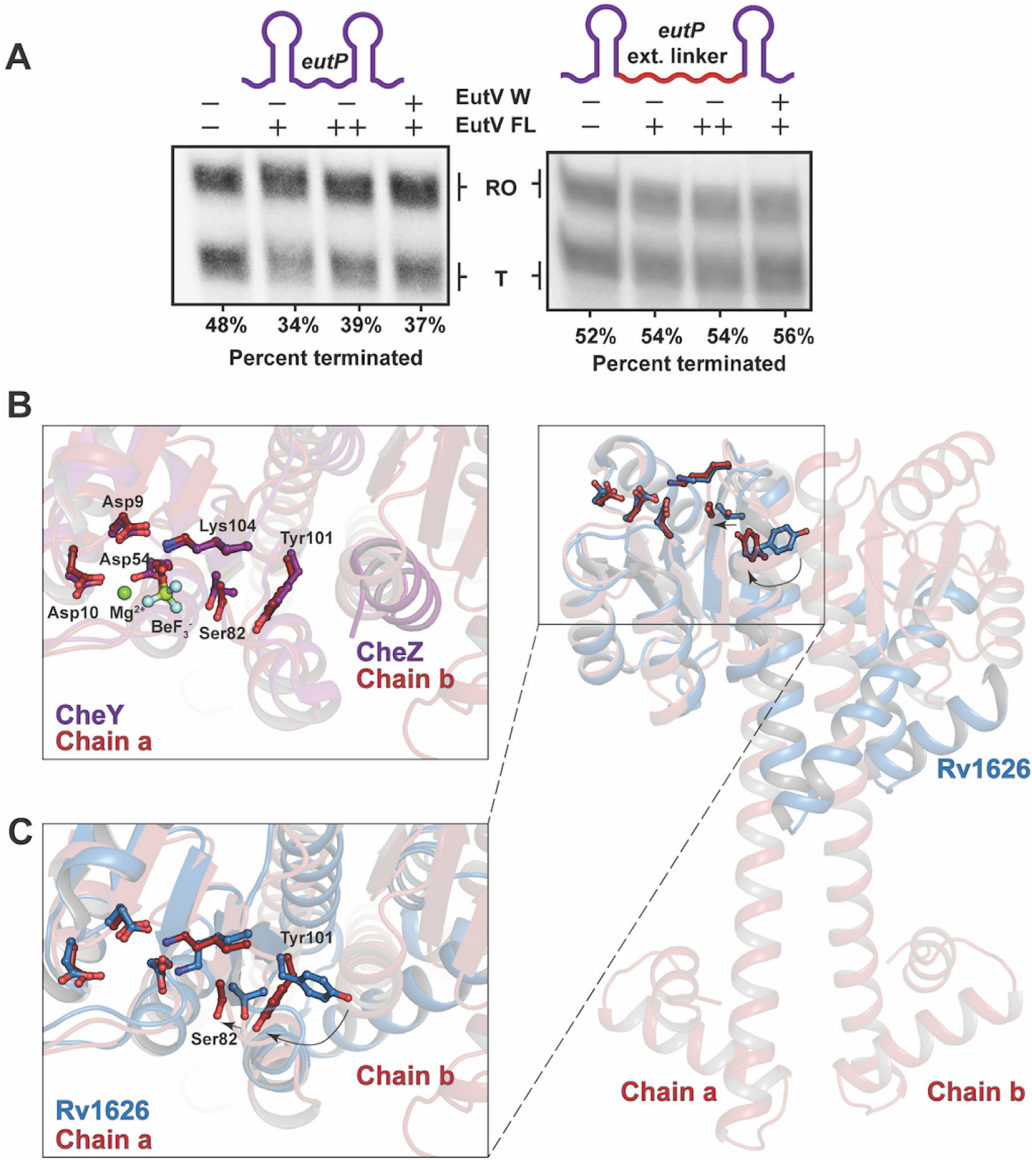
*In vitro* transcription antitermination and modelled phosphorylation of EutV. (**A**) *In vitro* transcription antitermination assays with the wild-type eutP template display a decrease in termination in the presence of EutV and phosphorylated EutV. This termination decrease is prevented when the inter-loop linker is extended. Representative gels of three independent replicates shown. (**B**) Chain a of the dimeric EutV structure overlaid with BeF_3_^–^ activated CheY (PDB: 2FMK) indicates the conserved residues involved in the phosphor-relay pathway are in the phosphor-activated orientation. (**C**) Overlay of the REC domain of chain a of EutV with the corresponding residues of the monomeric Rv1626 (PDB: 1S8N). Tyr101 is buried within the REC domain of EutV (relative to Rv1626) which allows chain b to associate with chain a through the α4–β5–α5 interface. Similar dimerization is sterically blocked in Rv1626.

*In vitro* transcription antitermination assays were carried out to determine if the ability of EutV to bind RNA *in vitro* correlates with *in vitro* transcription antitermination. Synchronized *in vitro* transcription assays utilized the T7A1 promoter and included a 162 nt stretch of the *eutP* leader region that encompassed the dual hexaloop motif. The addition of unphosphorylated EutV resulted in a concentration dependent decrease in transcription termination of ∼10% when 10 μM of EutV was added (Figure 5A, Supplementary Figure S18A). When the paired kinase EutW was also included, a similar level of antitermination was achieved using 10-fold less EutV. (Figure 5A, Supplementary Figure S18A) highlighting the well-established role of phosphorylation in EutV mediated antitermination (26,35). These results indicate that the EutV protein used throughout this study is capable of antitermination.

To confirm the importance of the linker length between the P1 and P2 hairpins, a 20 nt extension was added between the two hairpins of the previous construct. In the presence of this extended linker, neither EutV nor EutV-P showed antitermination (Figure 5A, Supplementary Figure S18B) highlighting the spatial requirement needed for successful antitermination, as previously reported (26).

### Monomeric EutV

Phosphorylation of EutV is known to promote dimerization and transcription antitermination (26,37). It stands to reason a construct incapable of dimerization should be deficient in both roles. Based on our crystal structures we generated an N-terminal EutV truncation (E140) that lacked the residues of the heptad repeat that are involved in coiled-coil formation but retained all residues that were identified for RNA binding (Supplementary Figure S19A). MALS analysis showed E140 to be monomeric at high concentrations (Supplementary Figure S19B) and showed a reduced affinity for RNA when assayed using EMSA (Supplementary Figure S19C). Initial binding occurring between 10 and 25 μM however, saturation was unable to be reached at concentrations exceeding 200 μM suggesting dimerization to be a precursor for stable EutV binding. Additionally, E140 did not show any activity in an *in vitro* antitermination assay (Supplementary Figure S19D).

## DISCUSSION

In the presence of ethanolamine, EutW phosphorylates EutV on a conserved Asp54 residue in the REC domain, promoting a shift in the homodimerization equilibrium (26). The dimeric RNA-free structure of EutV resembles the crystal structure of the ANTAR domain protein AmiR from *P. aeruginosa* (Supplementary Figure S20A and B). Like EutV, AmiR is a positive regulator of gene expression through a C-terminal ANTAR domain. However, AmiR itself is regulated by the direct interaction of AmiC with its N-terminal pseudo-REC domain that lacks residues required to accept phosphorylation from a TCS kinase (Supplementary Figure S20A). Thus, it has remained unclear how phosphorylation triggers dimerization of EutV.

Within the EutV structure, the conserved active site residues in the N-terminal REC domain are in a ‘phospho-activated’ state, as indicated by their position relative to the to the BeF_3_^–^ activated CheY/CheZ complex (Figure 5B) and the BeF_3_^–^ activated EutV bound RNA structure (Supplementary Figure S5). This positioning indicates that the dimeric structure of EutV observed in the crystal is representative of the biologically active phosphorylated dimer required for efficient antitermination (26,61–65). Despite its dimeric structure, EutV shares sequence identity (37%) and identical domain architecture with the antitermination protein Rv1626 from *M. tuberculous* (Supplementary Figure S21) (31,38). Like EutV, in the absence of phosphorylation Rv1626 exists as a monomer in solution and was crystallized in a monomeric state (Figure 5 C) but is thought to form an extended dimeric structure upon phosphorylation (31). The key differences between the Rv1626 and EutV crystal structures arise from the extended nature of the α5–α6 helix in EutV dimer relative to the monomeric Rv1626 (Figure 5C, Supplementary Figure S21). The active site residues in the REC domain of Rv1626 are in the non-phosphorylated state (Figure 5C), highlighted by an ‘outward’ facing Tyr111 (Tyr101 in EutV) that sterically inhibits a α4–β5–α5 dimer interface from forming as present in the EutV structure (Figure 5C). Given the high sequence identity between the two proteins, it is likely that the monomeric state of EutV seen in solution (Supplementary Figure S4) will adopt a similar compact conformation to Rv1626, representing the inactive state for antitermination. Comparison of the monomeric Rv1626 and the EutV dimer allows us to model the possible extension of the α5–α6 helices that a monomeric EutV would be required to undergo, upon phosphorylation of Asp54 (26,35), to form an extended state capable of dimerization through the coiled-coil domain (Figure 5C, Supplementary Figure S22).

A surprising revelation from the RNA bound structure of EutV was the orientation of the RNA hexaloops that contact each ANTAR domain. The RNA hexaloops face towards each other (Figure 2D, Supplementary Figures S6–S8) and this orientation prevents the EutV dimer from simultaneously contacting both hexaloops from a single RNA molecule (Supplementary Figure S8D). This positioning of the hexaloops is clearly biologically relevant given the conserved nature of the residues interacting with the RNA (Figure 4A) and the decrease in binding affinity seen when these residues are mutated to alanine (Figure 4B). Interestingly, Met172 is essential for RNA binding (Figure 4B), serving to correctly position the hexaloop on α7 by acting as a hydrophobic plug (Figure 3F). It may also act as a size determinant for the RNA loops that EutV is able to bind. Given its hydrophobic nature and proximity to Tyr164, Met172 may act to prevent smaller and more common RNA loops, such as tetraloops, from erroneously binding EutV. Methionine residues are not typically associated with RNA:protein interactions and, to our knowledge, this is the first example of this residue being obligatory for RNA binding. This finding may represent a novel mode of protein:RNA secondary structure interaction.

Position 1 and 4 in the hexaloops of the dual hairpin binding motif are conserved as adenosine and guanine bases respectively (A1 and G4) (Figure 2A and B) (26). The X-ray crystal structure has revealed the critical role G4 plays in antitermination through π-π stacking with Tyr164 (Figure 3A–C). However, A1 does not make specific contacts with EutV in the crystal structure and therefore its conserved nature is not likely due to direct interaction with EutV but rather to assist in the RNA fold. Hexaloops structures are capable of folding into pseudotriloops through cross-loop base pairing (66,67). Given the requirement for a G4 of the hexaloop to flip outward to interact with EutV, it is possible position 1 is conserved as an adenosine to prevent the formation of pseudotriloops that may form if other bases were present. The Met172 hydrophobic plug may also contribute to preventing pseudotriloops from folding.

The structure of the EutV bound to RNA identified two binding sites, one at each ANTAR domain of the dimer. Comparison of the REC domains and the ANTAR domains of each chain in both the RNA free and bound structures indicate no changes in the secondary structure of these domains upon RNA binding (Supplementary Figure S23). Nevertheless, there is a break in symmetry of the homodimer upon RNA binding due to a larger flex in the coiled-coil of chain b, between Ile139-Glu141, than for chain a (Supplementary Figure S12). This asymmetric flex facilitates the interaction between the ANTAR domains of both chain a and chain b and the P2 hexaloop (Figure 4C) and prevents the ANTAR domain of chain a forming the same set of interactions with the P1 hexaloop (Figure 3D, Supplementary Figure S10). Given that the RNA bridges between ASUs and makes a crystal contact with a neighbouring ANTAR domain (Supplementary Figure S8), it is possible that this asymmetric flexing is a crystallization requirement and does not represent a biologically relevant interaction. However, this is unlikely for two reasons: first, in the absence of any movement in chain a, the RNA-binding residues on α6-α7 of the chain b ANTAR domain would be occluded (Supplementary Figure S24). Second, the conserved nature of the residues (Figure 4A) that are positioned to bind RNA, as a consequence of the large flex in chain b, suggest a biological importance that was confirmed by alanine mutagenesis and SPR (Figure 4A–C, Supplementary Figure S14) which is consistent with a recent mutagenesis study of NasR that identified similar residues to be important for RNA binding (34). Of particular interest was the 5-fold and 8-fold reduction in affinity of the K143A/K147A and R142A constructs respectively. Due to the asymmetric flex, these protein:RNA interactions can only occur between chain b and P2, not chain a and P1. In summary, both chains of the dimer are unable to make the same set of interactions with the RNA hairpins at the same time and furthermore, given the RNA binding orientation, there is no plausible way both hairpins of the dual hexaloop motif can contact the dimer at the same time. This raises the possibility that the protein, in its biological role, does not contact both hairpins of the dual hairpin motif at the same time.

Within the crystal, EutV contacts two RNA hexaloops indicating the presence of two, albeit different, RNA binding sites (Figure 3, Supplementary Figure S10). These hexaloops do not come from the same RNA molecule and raises the possibility that during transcription, a EutV dimer may contact two RNA hairpins from the nascent RNA of two separately transcribing RNA polymerases. This scenario is unlikely, given the rapid timescale of transcription: once the T-loop has folded, it cannot be remodelled and transcription will terminate (2). SPR studies conducted on alanine mutants confirmed that each RNA binding residue of EutV identified between the ANTAR domain of chain b and the P2 hexaloop (Figure 3A-B) plays a role in binding RNA *in vitro* (Figure 4B). Upon RNA binding, the asymmetric flex of the coiled-coil domains of EutV results in a differing set of protein:RNA interactions between chains. Given it is unlikely for the EutV dimer to bind two hexaloops in *trans* and the inability of the dimer to bind both hexaloops simultaneously, it is suggested that the second RNA binding site (between the ANTAR domain of chain a and P1) (Figure 3C) provides a snapshot of a transitional binding state between a single ANTAR domain (of the dimer) and an RNA hexaloop that likely occurs prior to the full protein:RNA interactions seen between both ANTAR domains and a single hexaloop (Figure 3A and B) (Supplementary Movie S1).

This role in the initial hexaloop binding is supported by evidence that the truncated monomeric EutV construct (E140), that retains all residues required for RNA binding, maintains the ability to bind the dual hexaloop motif, albeit with lower affinity than the full length (Supplementary Figure S19C). The smeared nature of the bound fraction, relative to the phosphorylated full length EutV (compare Supplementary Figure S15B and Supplementary Figure S19C) suggests a weaker interaction that can be explained by the loss of interactions that only between a dimeric EutV and RNA hairpin (Figure 3D). This decrease in binding affinity in a monomeric EutV is consistent with previously published EMSA experiments (26).

If the EutV dimer only binds a single RNA hairpin, the question still remains: why are both hairpins required for antitermination? Furthermore, why is dimerization a conserved mechanism if both ANTAR domains do not make similar contact with both RNA hexaloops simultaneously (26,35)? It is plausible that two EutV dimers bind each hexaloop independently. EMSA using EutP RNA confirms a dual binding event in solution, suggesting a 2:1 binding stoichiometry (Supplementary Figure S15) and is consistent with the binding observed in the crystal structure (Figure 2D, Supplementary Figure S8). However, as the upstream hairpin (P1) does not overlap with the T-loop, independent binding of both dimers is incompatible with the obligatory requirement for both hexaloops to be present for EutV mediated antitermination *in vivo* (26).

The possibility that binding of one EutV dimer to the upstream hairpin results in a remodelling of the P2 hairpin that facilitates a rapid binding of a second EutV dimer cannot be excluded. Likewise, the proximity of the 5′- and 3′-ends of the RNA hexaloops at one interface between neighbouring ASUs within the crystal lattice may indicate a biologically important EutV tetramer complex containing two EutV dimers and a single dual hexaloop RNA motif (Supplementary Figure S25). However, the only interface (846 Å^2^) within a tetramer complex lies between the REC domain of one EutV dimer (chain a) and the ANTAR domain of another EutV dimer (also chain a) suggesting such a binding mode would not be conserved in ANTAR proteins lacking a REC domain (Supplementary Figure S25B). Furthermore, the residues involved in this interface have been implicated in either the correct folding of the ANTAR domain three-helical bundle or the phosphorylation of the REC domain, indicating their conserved nature may be a result of these functions, and not the formation of a larger complex (Supplementary Figure S25C) (24,68).

In the context of a transcribing polymerase, the P1 and P2 hexaloops are transcribed successively, not simultaneously. In a similar fashion, EutV binding to each hexaloop may occur successively and therefore not require both hairpins to be transcribed before binding occurs. A successive binding model would require EutV be capable of binding a single RNA hexaloop, implies a spatial constraint must exist between each of the two RNA hexaloops, and that successful antitermination would only occur with a dimeric protein. SPR assays revealed EutV binds each isolated P1 and P2 hexaloop with similar binding affinity (Figure 4D–G). Furthermore, *in vitro* transcription assays demonstrate that extending the linker region between hexaloops by 20 nt was sufficient to abolish EutV mediated antitermination (Figure 5A). This is consistent with a previous study that indicated any modification to the inter-hairpin length, beyond the distance that exists within the *eut* operon (Supplementary Figure S3B) inhibits *in vivo* antitermination and suggests a strong spatial constraint exists for the inter-hairpin distance. Finally, a monomeric EutV construct E140 (Supplementary Figure S19A and B) that retained RNA binding (Supplementary Figure S19C) was unable to promote antitermination *in vitro* (Supplementary Figure S19D). The delineation of RNA binding and *in vitro* antitermination, in the monomeric E140 construct, suggests only a dimeric protein is capable of antitermination and is highly indicative of a successive binding mechanism, rather than a simultaneous one.

**Figure 6.**
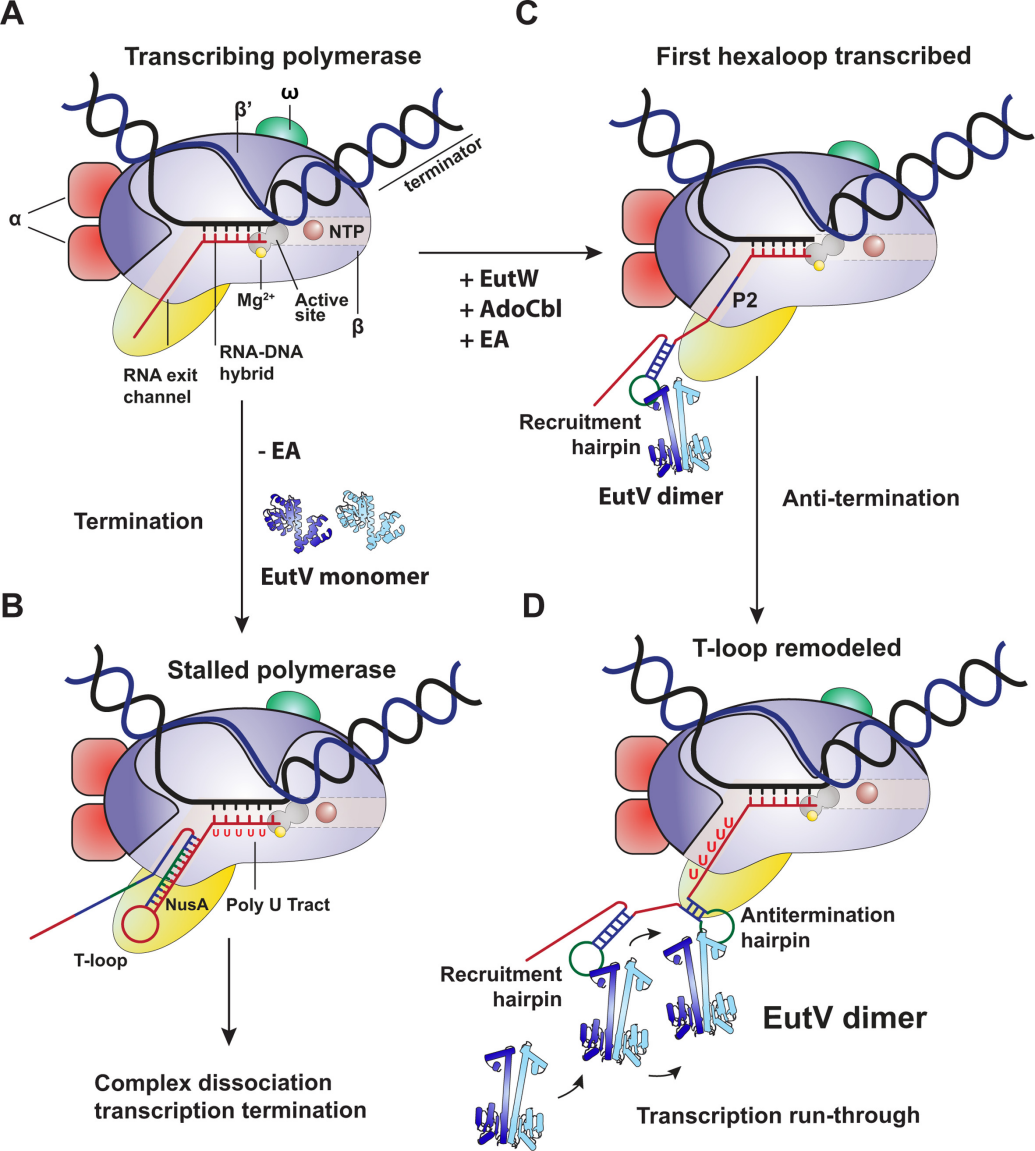
Proposed model for EutV antitermination. (**A**) Core RNA polymerase (RNAP) (in bacteria composed of an α dimer, a β subunit, a β′ subunit and an ω subunit) is bound to the DNA duplex composed of the template strand (black) and the non-template strand (blue), and the nascent RNA (red). (**B**) Stalled RNAP on poly-U tract. Intrinsic T-loop formation within the RNA exit tunnel is stabilized by NusA (yellow). (**C**) In the presence of EutW, AdoCbl and ethanolamine dimeric EutV binds the first hexaloop (P1) of the dual hairpin motif before cycling to the (**D**) second hexaloop (P2) as transcription continues.

We propose a revised model for EutV mediated antitermination in the context of the transcribing RNAP that most reasonably fits our observations and those present in the literature. In the absence of ethanolamine (EA), EutV remains unphosphorylated and monomeric, leading to intrinsic termination of transcription at each T-loop (Figure 6A and B). When present, EA stimulates EutW phosphorylation of EutV, resulting in an increased sampling of the dimeric state as described in (26,35,37). Dimeric EutV binds the P1, or recruitment hairpin, first (Figure 6C), bringing it into proximity to the RNAP. This initial contact, as shown by the interaction between chain a and P1 in the crystal structure, is followed by a large flex in the same ANTAR domain that allows K143/K147 of the second ANTAR domain to bind to the same hexaloop, as represented by the interaction between chain b and P2 in our structure (Supplementary Movie S1). Full binding of chain b to a single hairpin places the second unbound ANTAR of the EutV dimer in close proximity to the RNA exit tunnel of the transcribing polymerase. As the P2, or antitermination hairpin, is transcribed it folds in proximity of the EutV dimer bound to the recruitment hairpin and may facilitate the transition of the EutV dimer from the recruitment hairpin to the antitermination hairpin (Figure 6C and D). Providing the EutV dimer stabilizes the antitermination hairpin long enough for the polymerase to by-pass the poly-U tract, transcription will continue unabated. The recruitment hairpin may be bound by a second EutV dimer, after the first dimer has transitioned to the antiterminator hairpin, which would likely prevent a backwards transition of a EutV dimer. This model provides the most rational explanation for both the inability of a single EutV dimer to contact both hexaloops from a single RNA molecule, as seen in our crystal structure, and the requirement for the P1 hexaloop to be present to facilitate *in vivo* antitermination (26). Furthermore, both ANTAR domains of the EutV dimer are utiliszed, although not simultaneously, providing the rationale for dependence on EutW, and thereby EA-induced EutV phosphorylation/dimerization, for antitermination *in vivo* (26). This model also explains the 2:1 protein/RNA stoichiometry seen in both the crystal structure (Figure 2D, Supplementary Figure S8) and EMSA (Supplementary Figure S15B) with the potential second dimer binding to the recruitment hairpin, after the first dimer has transitioned to the antitermination hairpin. Finally, this model explains the spatial constraint that is applied to the linker between hairpins of the dual hexaloop motifs of the *eut* operon (Figure 5A, Supplementary Figure S3) (26).

As dimeric EutV is unable to bind both hexaloop simultaneously we have redefined the minimal ANTAR domain target motif to a single hexaloop motif. This allows for potential single hexaloop ANTAR binding sites to exist within the bacterial genome when the time constraint of a transcribing polymerase is absent. Indeed in *E. faecalis* this may be true for the sequestration of EutV by the small non-coding EutX RNA (*rli55* in *L. monocytogenes* (69)) in the absence of essential cofactors required for EA metabolism (Supplementary Figure S26). Inspection of the EutX sequence identified an additional single hexaloop, with a G4 nucleotide, in addition to the previously described dual hexaloop motif (70). Furthermore, recent work describing the presence of RNA stemloops that overlap with ribosome binding sites and 5′-UTR of transcripts in *Mycobacterium tuberculosis* do not always obey the classical dual hexaloop motif (39). Further bioinformatic analysis and experimental work is required to determine to what degree single hexaloop motifs play in ANTAR domain function and what novel processes they may regulate.

## DATA AVAILABILITY

Atomic coordinates and structure factors for the EutV crystal structures are available from the Protein Data bank under accession numbers 6WSH (EutV alone) and 6WW6 (EutV:RNA bound).

## Supplementary Material

gkac074_Supplemental_Files

## Notes

Present address: James L. Walshe, Department of Molecular Biology, Max Planck Institute for Multidisciplinary Sciences, Göttingen, Germany.
