# Supplementary material for: Structural characterization of the ANTAR antiterminator domain bound to RNA
Source: Nucleic Acids Res. 2022 Feb 12;50(5):2889–904. doi: 10.1093/nar/gkac074 (PMC8934654; doi:10.1093/nar/gkac074)
Supplement: gkac074_Supplemental_Files [file gkac074_supplemental_files.zip › 220112_Walshe_SupplimentaryFigures_FINAL.pdf]

Supplementary Materials

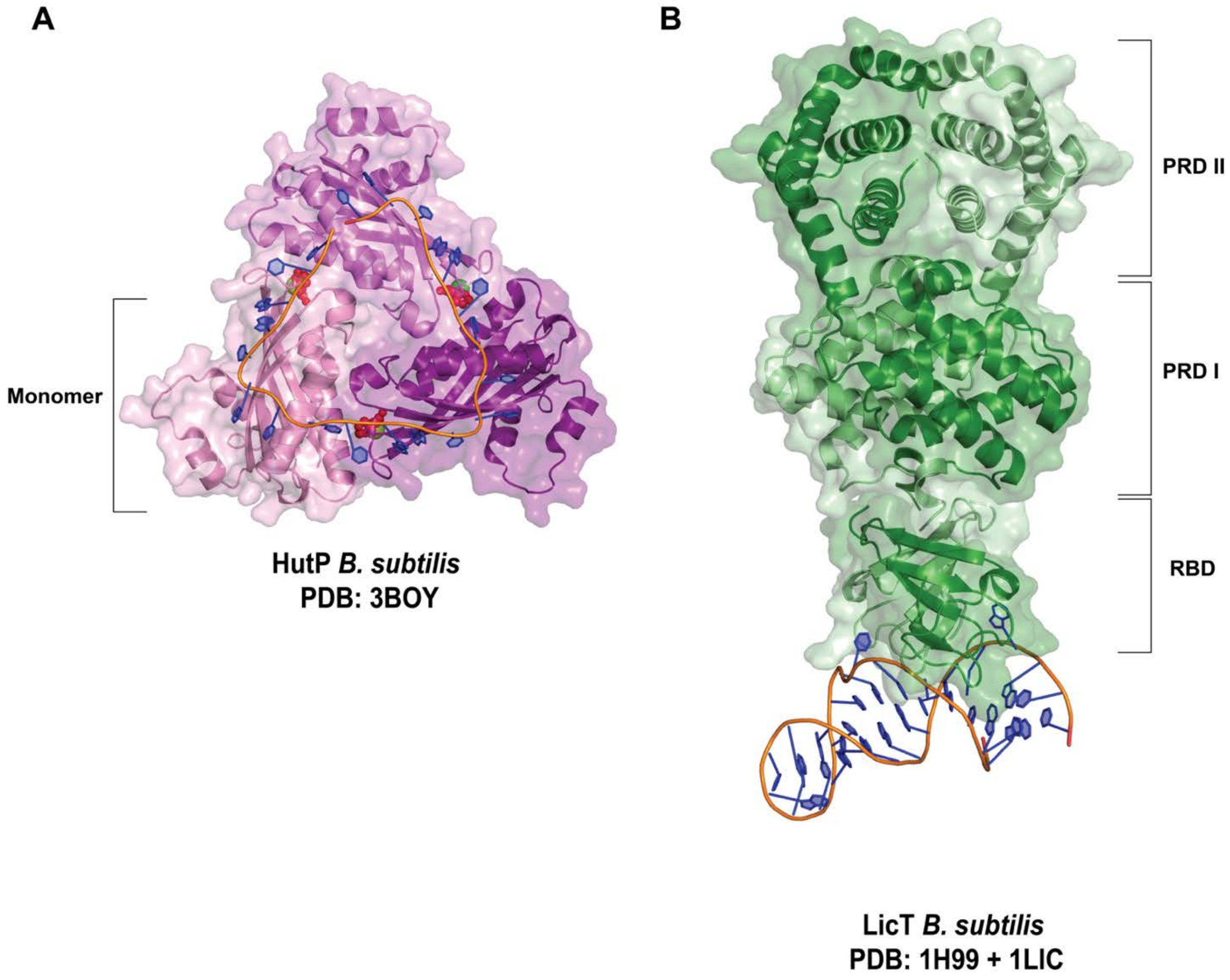

Supplementary Figure S1. PyMOL cartoon and surface representation of two characterised RNA binding antiterminators. (A) HutP (*B. subtilis*) hexamer bound to its cognate RNA sequence (PDB: 3BOY). Histidine ligands, shown as small red spheres, and Mg^{2+} ions, as large green spheres, bind at the trimer interfaces, supporting biochemical evidence that proper HutP function requires these ligands. NAG triplets bind to each monomer. (B) Modelled complex of N-terminal LicT (*B. subtilis*) bound to RNA (PDB:1LIC) and C-terminal LicT PRD domains (*B. subtilis*) (PDB 1H99). Phosphorylation events in the PTS regulation domain (PRD) II and PRD I dictate dimerisation of LicT, which facilitates binding to a structured RNA element through the RNA binding domains (RBD). Ligands are presented as small red spheres, RNA bases as blue rings and the phosphate backbone as an orange tube.

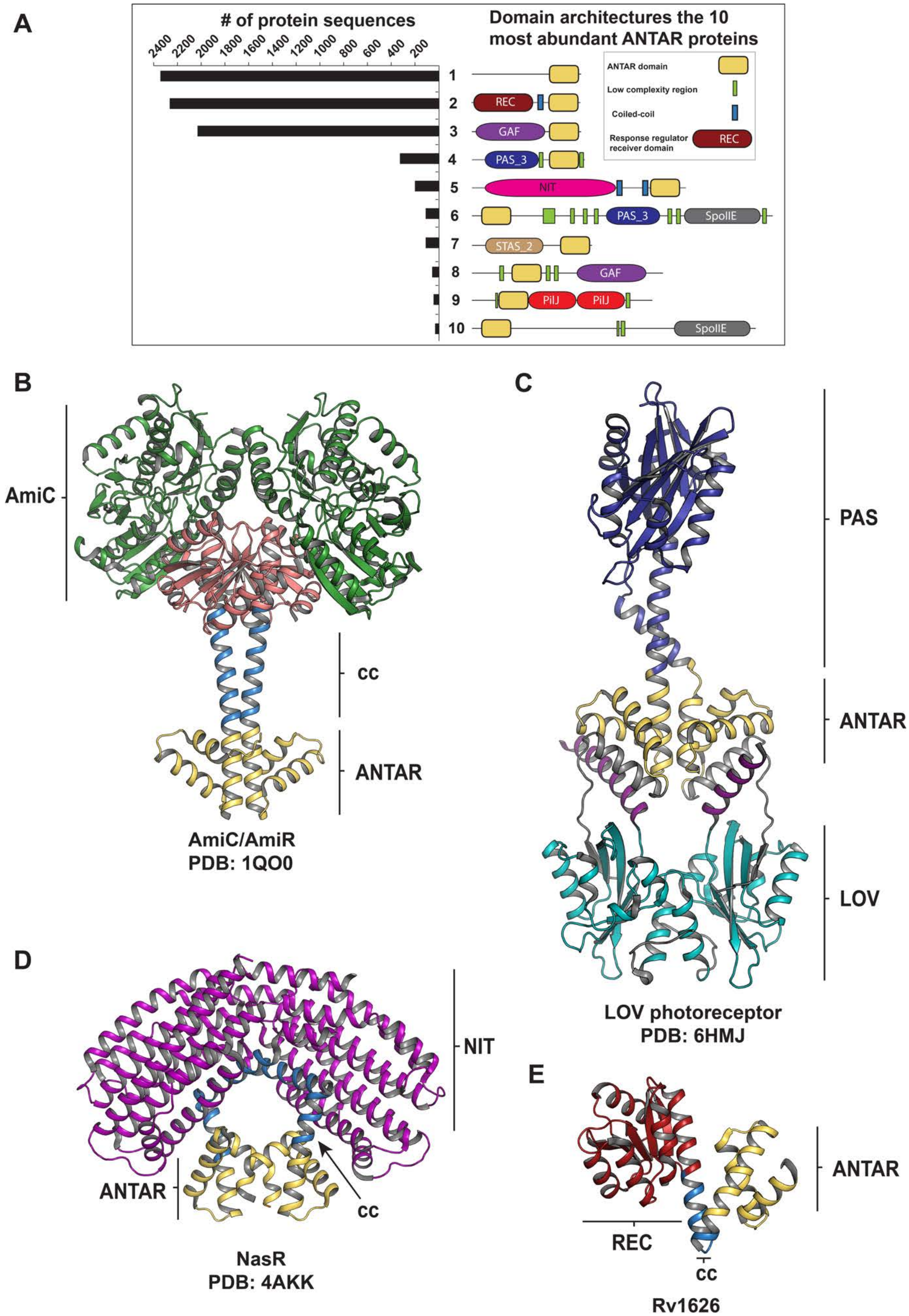
A
# of protein sequences
2400
2200
2000
1800
1600
1400
1200
1000
800
600
400
200
Domain architectures the 10 most abundant ANTAR proteins
ANTAR domain
Low complexity region
Coiled-coil
Response regulator receiver domain
REC
1
2
3
4
5
6
7
8
9
10
GAF
PAS_3
NIT
SpoIIE
STAS_2
PilJ
B
AmiC
cc
ANTAR
AmiC/AmiR
PDB: 1QO0
C
PAS
ANTAR
LOV
LOV photoreceptor
PDB: 6HMJ
D
NIT
ANTAR
cc
NasR
PDB: 4AKK
E
ANTAR
REC
cc
Rv1626
PDB: 1S8N

Supplementary Figure S2. Structures of the four known ANTAR domain proteins and distribution of ANTAR domain proteins according to their domain organisation (A) Bar graph showing the number of ANTAR proteins that contain the domain organisation shown schematically on the right hand side of the figure. Figure adapted and updated from (1). (B-E) ANTAR domains coloured yellow and predicted coiled-coil domains in sky-blue. All structures are in an inactive state. (B) The *P. aeruginosa* amidase operon anti-terminator AmiR in complex with the negative regulator AmiC (green) (PDB 1QO0). AmiR forms an intimate dimer through a coiled-coil domain and pseudo-receiver domain (salmon). (C) Light-oxygen-voltage (LOV) photoreceptor PAL from *N. multipartite.* PAS and LOV domains colored in blue and cyan respectively (D) nasREDCBA operon anti-terminator protein NasR from *K. oxytoca* (PDB 4AKK). Nitrate/nitrite sensing (NIT) domain colored in purple. (E) Putative anti-terminator protein Rv1626 from *M. tuberculosis* (PDB 1S8N). Two component response regulator (RR) domain colored in red.

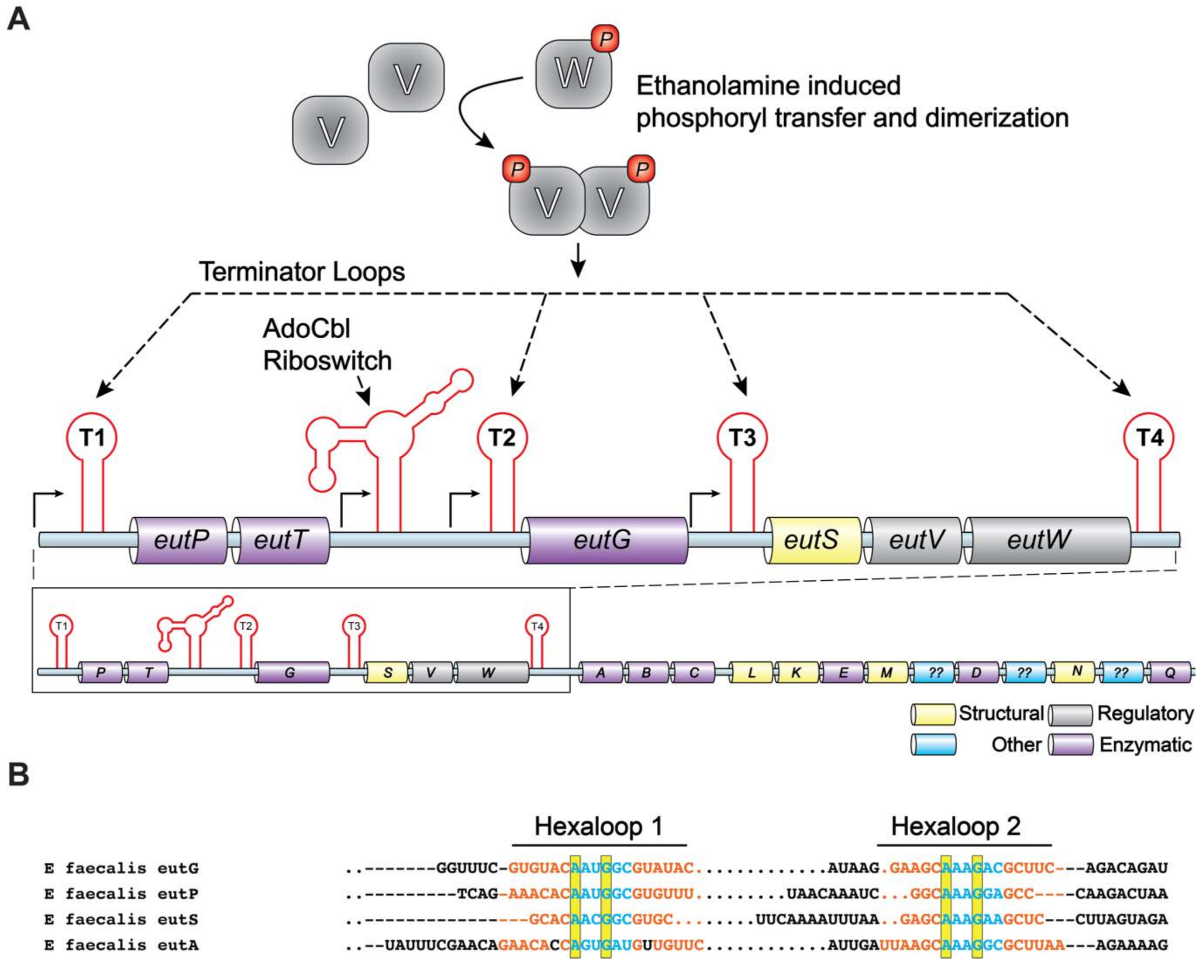

Supplementary Figure S3. Schematic representation of genes and regulatory elements within the *eut* operon from *E. faecalis*. Genes within the *eut* operon allow for the efficient catabolism of ethanolamine and provide host bacteria with a source of carbon and/or nitrogen. Histidine kinase EutW phosphorylates EutV in the presence of ethanolamine inducing dimerisation (A) Four intrinsic terminator loops and a riboswitch (shown in red) facilitate the regulation of expression of genes within the *eut* operon. Dimeric EutV disrupts the formation of the intrinsic terminator loops. (B) Primary sequence of the four dual hexaloop antiterminator elements from the *eut* operon. Bases involved in stem formation shown in orange, hexaloop bases in blue and with conserved bases at position 1 and 4 of the hexaloops highlight in yellow boxes.

A

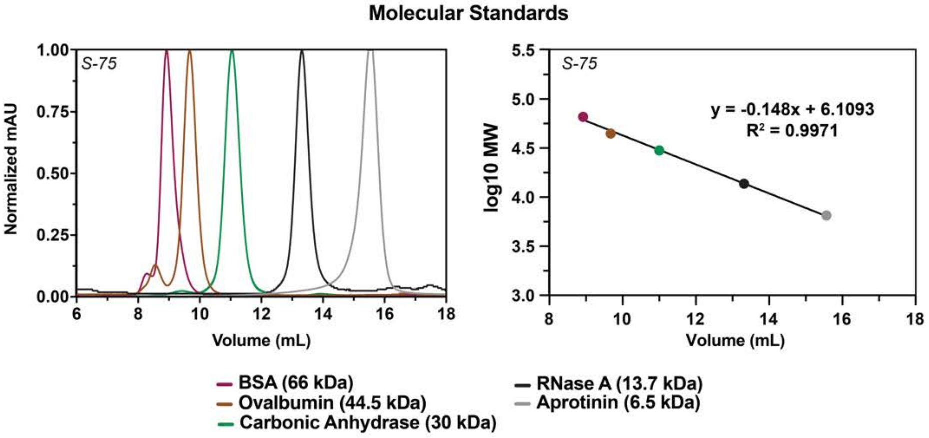

B

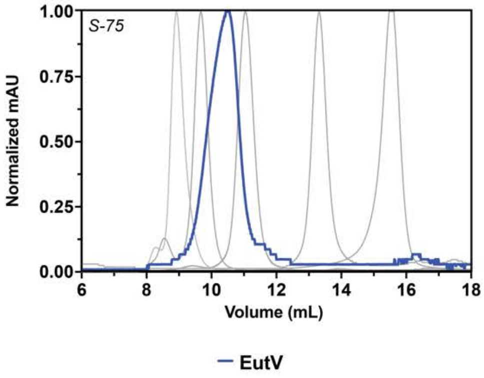

	MW (kDa)	Analytical SEC
EutV FL	21.3	≈ 36

C

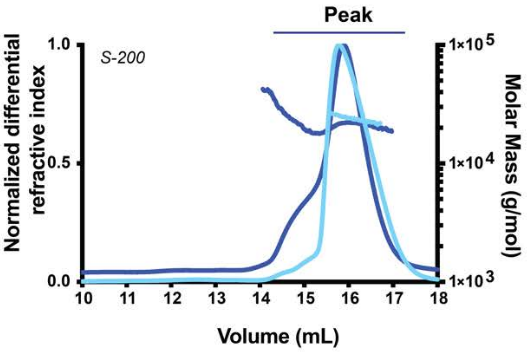

	MW	MALS
EutV 100 µM	21.3 kDa	21.9 ± 0.6 kDa
EutV 25 µM	21.3 kDa	24.9 ± 0.8 kDa

D

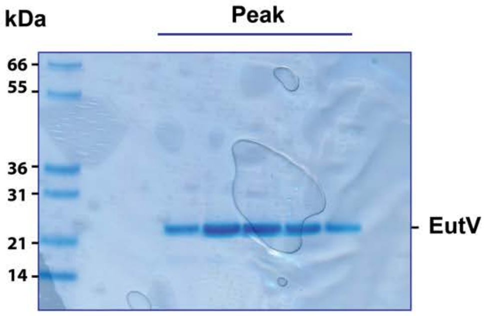

Supplementary Figure S4. Analytical SEC and SEC-MALS analysis of EutV. (A) Normalised mAU traces for the elution of five globular standard proteins from a Superdex75 10/300 column (Cytiva) and the associated calibration curve showing $\log_{10}$ MW on the *y*-axis and versus elution volume on the *x*-axis. (B) Normalised mAU traces for full-length EutV (blue), overlaid over the molecular standard traces (grey) and the molecular weight estimate calculated by analytical SEC. (C) Size exclusion chromatogram showing normalised differential refractive index on the left y-axis and

molecular mass estimates displayed as a scatter plots over each peak (right-hand y-axis). EutV was injected at either 25 μM (cyan trace) or 100 μM (blue trace) and separated on a Superdex200 10/300 Increase column. MALS analysis of the peaks reveals the protein is largely monomeric (24.9 ± 0.8 kDa) in solution when injected at 25 μM, although a larger molecular weight species is also present when a higher concentration of protein (100 μM) used. (D) SDS-PAGE showing the higher molecular weight species is not caused by a contaminating protein, suggesting that a concentration dependant dimer is present.

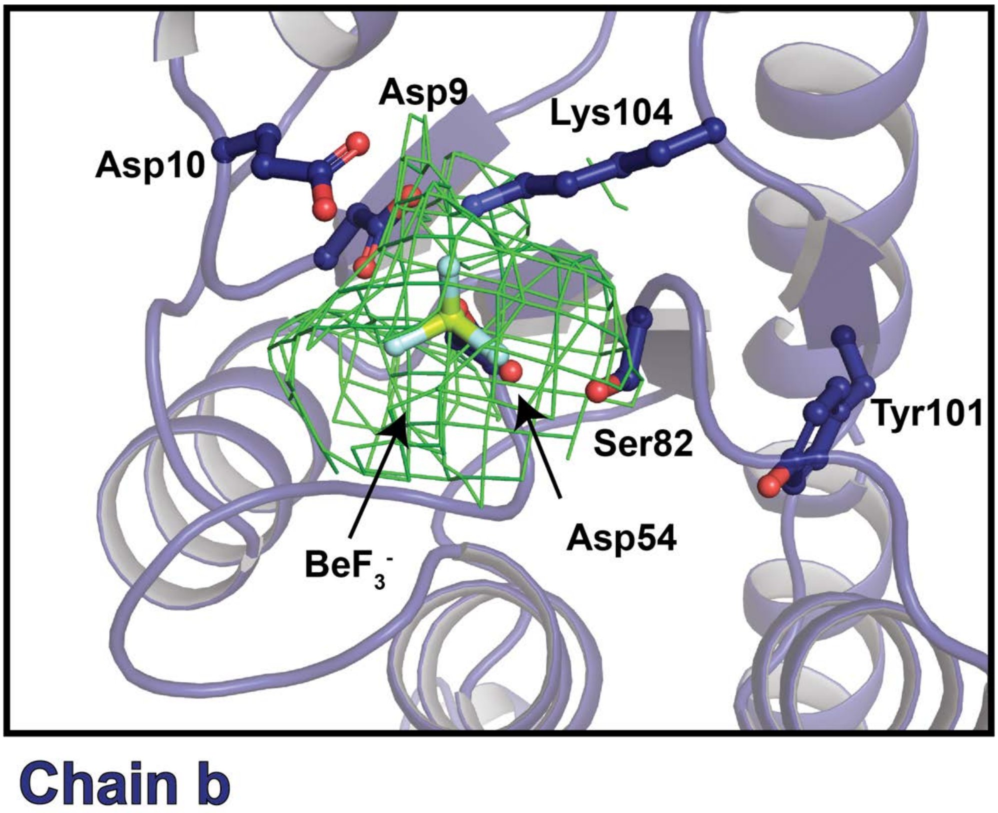

Supplementary Figure S5. Unmodeled electron density for BeF_3^-. Electron density map of EutV:EutP RNA dataset after molecular replacement and rigid body refinement using the CheY-like and ANTAR domains of EutV. F_o-F_c maps are contoured to at 3 σ. The N-terminal domain of EutV chain b is coloured blue and the residues involved in phosphorylation shown as sticks.

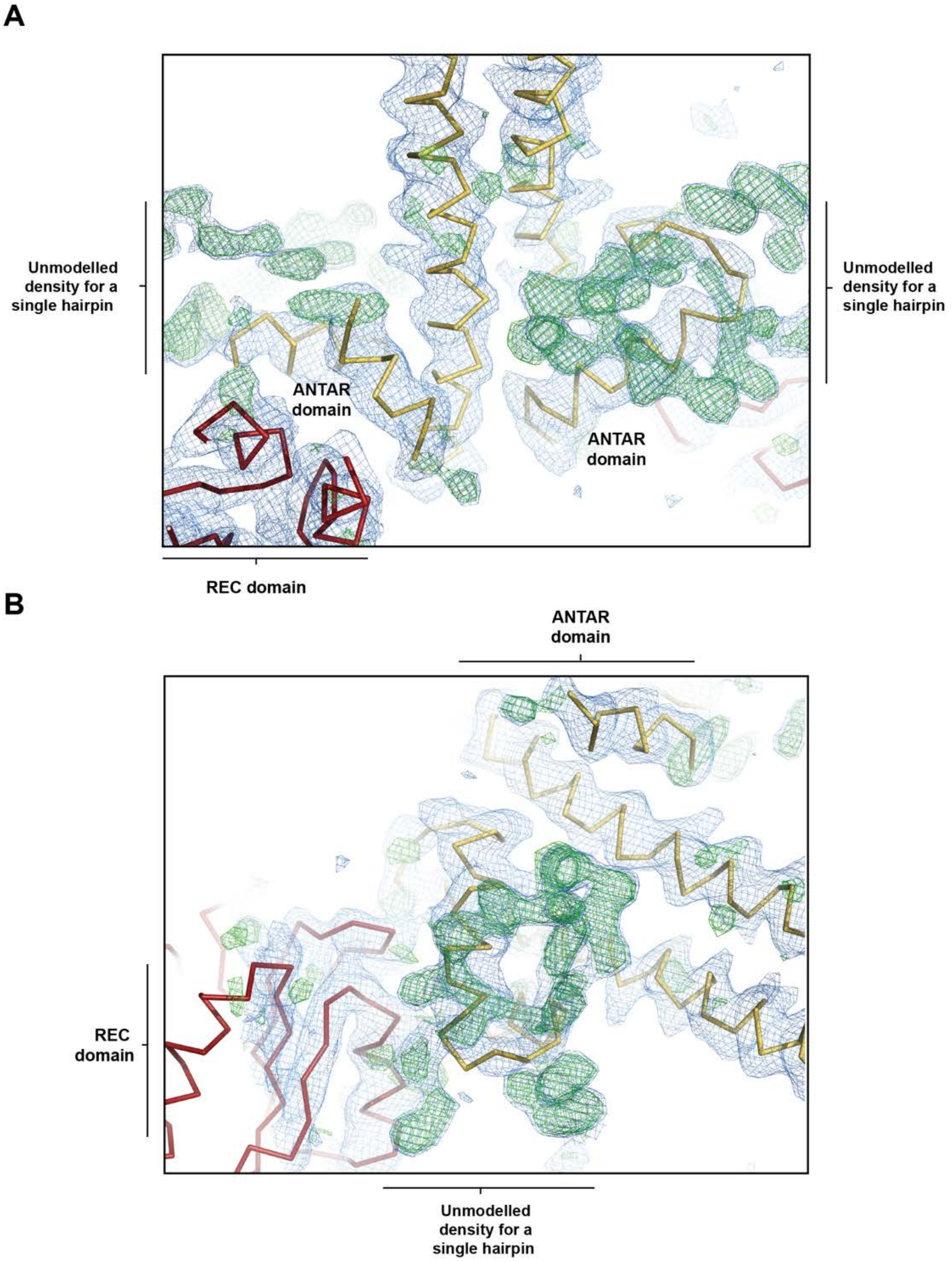

Supplementary Figure S6. Unmodeled electron density for RNA hairpins. Electron density map of EutV:EutP dataset after molecular replacement and rigid body refinement using the CheY-like and ANTAR domains of EuV. $2F_o$-F_c and F_o-F_c maps are contoured to at 1.5 σ and 3 σ respectively. The N-terminal CheY-like domain and C-terminal coiled coil/ANTAR domains are shown in red and yellow respectively.

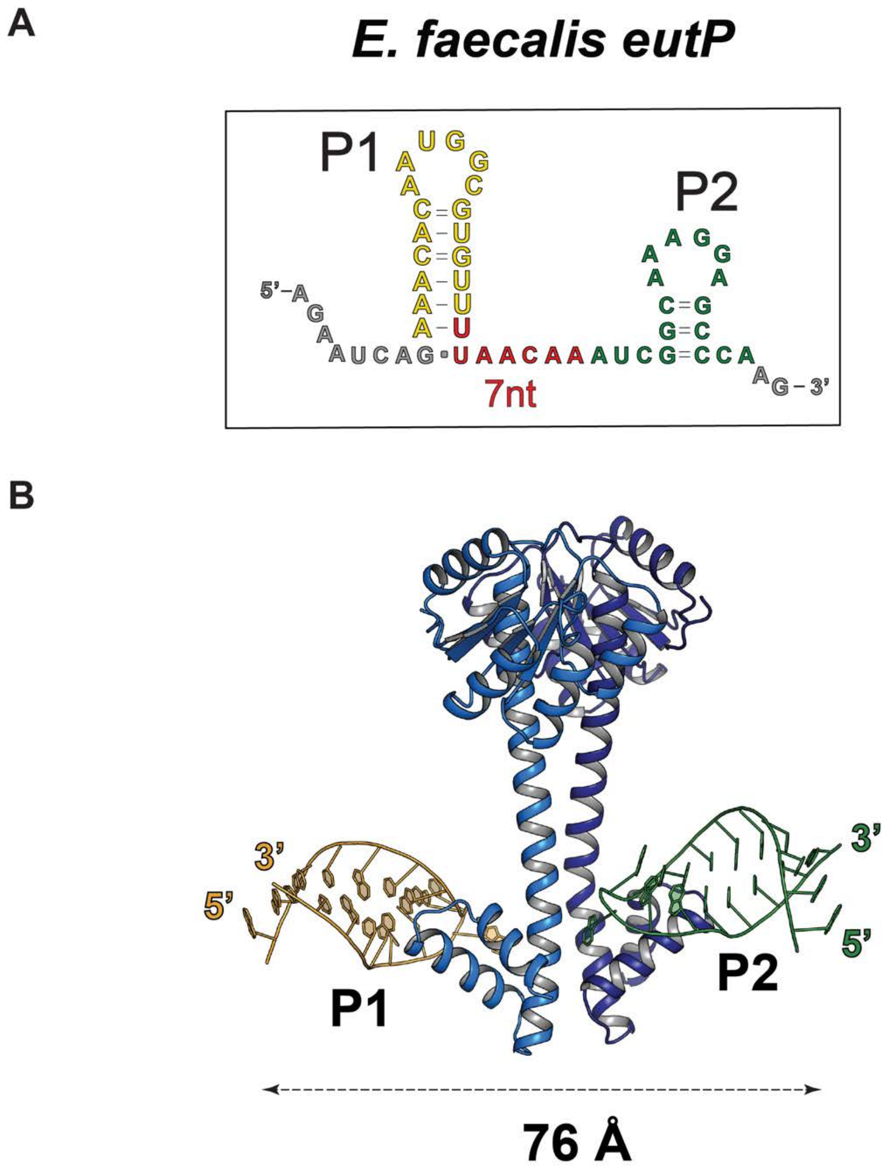

Supplementary Figure S7. Distance between model P1 and P2 hexaloop hairpins. (A) Primary sequence of the 51-nt RNA used for co-crystallisation with EutV. Yellow and green bases represent the successfully modelled P1 and P2 loops respectively. Bases in grey represent the unmodeled 5' and 3' ends and in red for the unmodeled linker. (B) Cartoon representation showing the modelled P1 and P2 loops with the distance between the 5' end of each indicated.

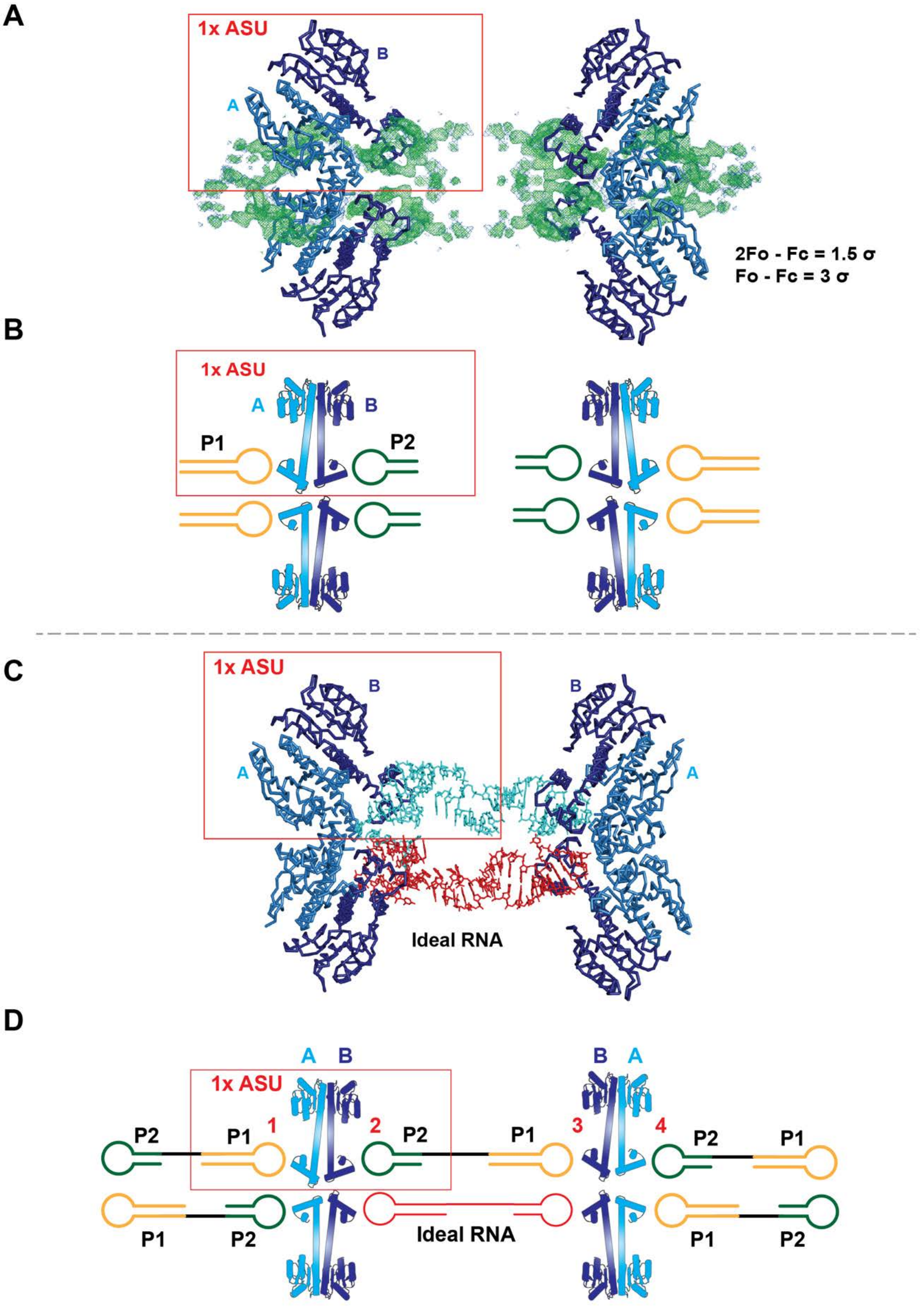
A
1x ASU
A
B
2Fo - Fc = 1.5 σ
Fo - Fc = 3 σ
B
1x ASU
A
B
P1
P2
C
1x ASU
B
A
B
A
Ideal RNA
D
A B
B A
1x ASU
1
2
3
4
P2
P1
P2
P1
P2
P1
P1
P2
Ideal RNA
P1
P2

Supplementary Figure S8. Crystal packing of the RNA bound structure. Each asymmetric unit (red box) is composed of one dimer of EutV and electron density for two RNA hairpins. Four asymmetric units (ASU) are shown in (A), the orientation of the hairpins relative to the EutV chains is shown schematically in (B). (C) Idealised *euP* RNA model (cyan and red) manually fitted between two ASUs. RNA generated with *RNAComposer* (2). (D) Schematic diagram of the EutV:*eutP* RNA crystal lattice. The four possible orientations of EutP RNA contacting the EutV dimer are numbered in red. Chain A and B of the protein is coloured in cyan and dark blue respectively. P1 and P2 hexaloops are coloured in green and cyan respectively. $2F_o$-F_c and F_o-F_c maps contoured to 1.5 σ and 3 σ respectively.

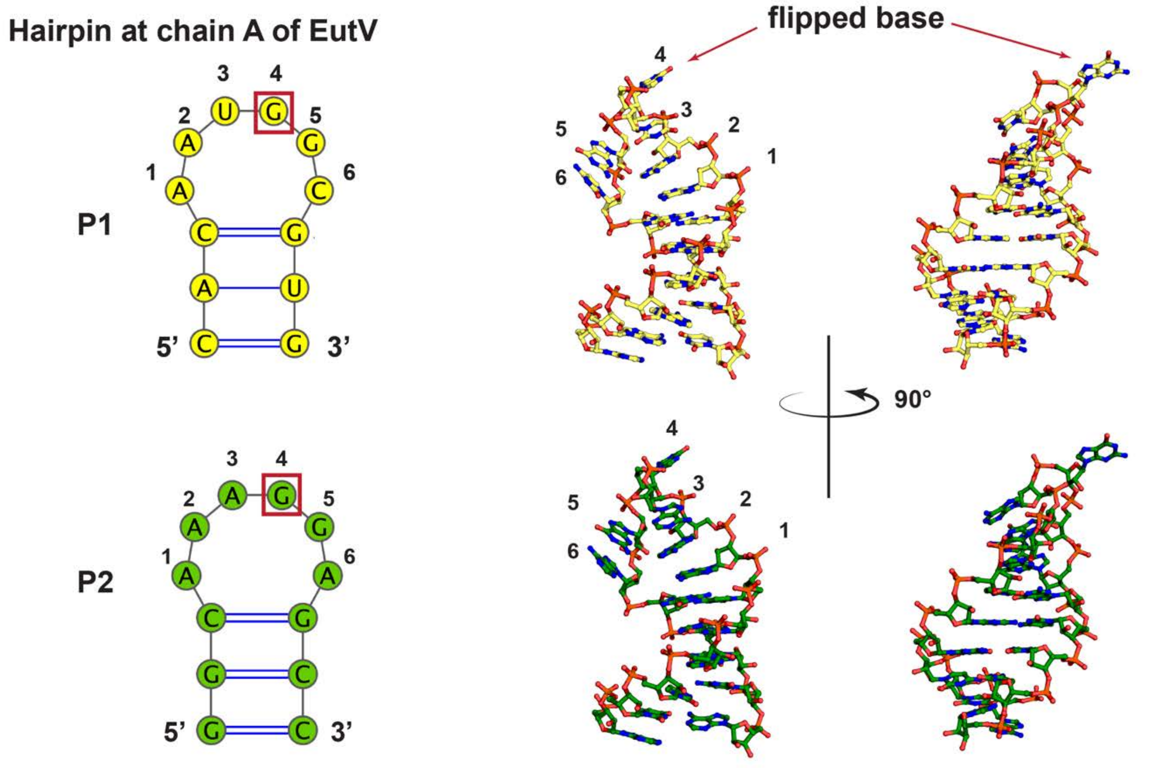
Hairpin at chain A of EutV
P1
P2
5'
3'
flipped base
90°

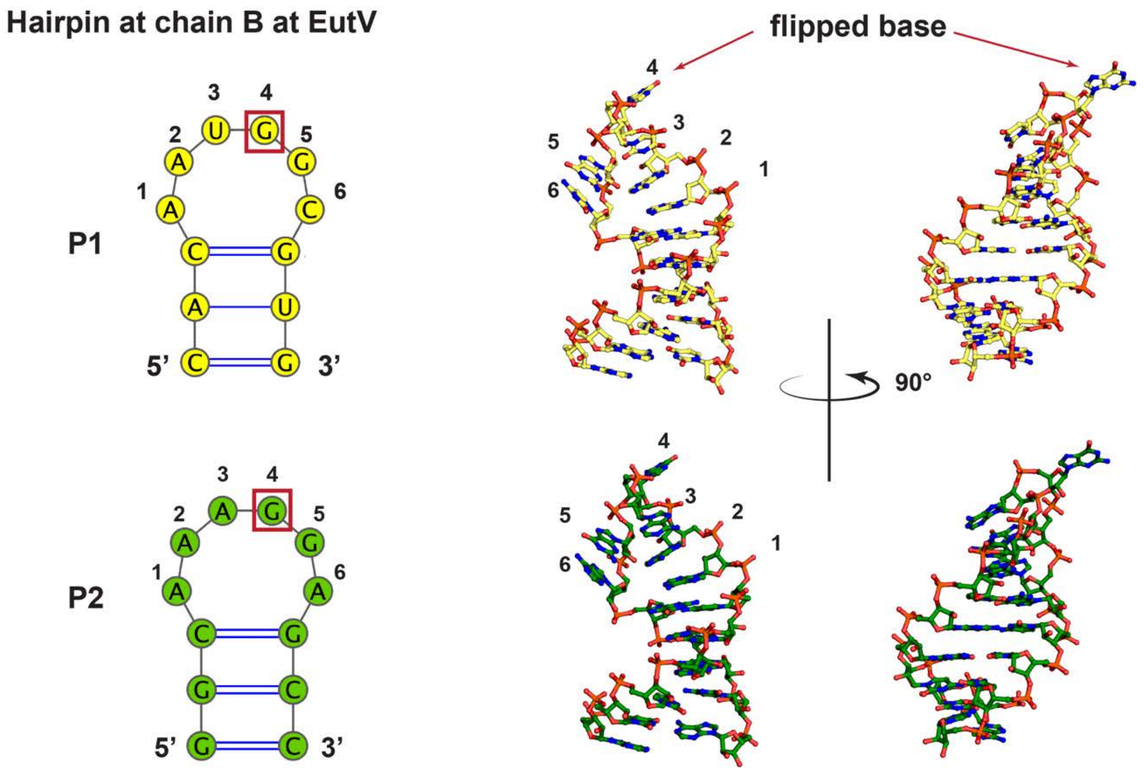
Hairpin at chain B at EutV
P1
P2
5'
3'
flipped base
90°

Supplementary Figure S9. Cartoon representation of the two modelled hairpins (P1 and P2) at either ANTAR domain of the EutV dimer. After dual-occupancy refinement in *Phenix.refine* (3), both P1 and P2 hairpins and the ANTAR domain of each chain of EutV refined to near identical positions.

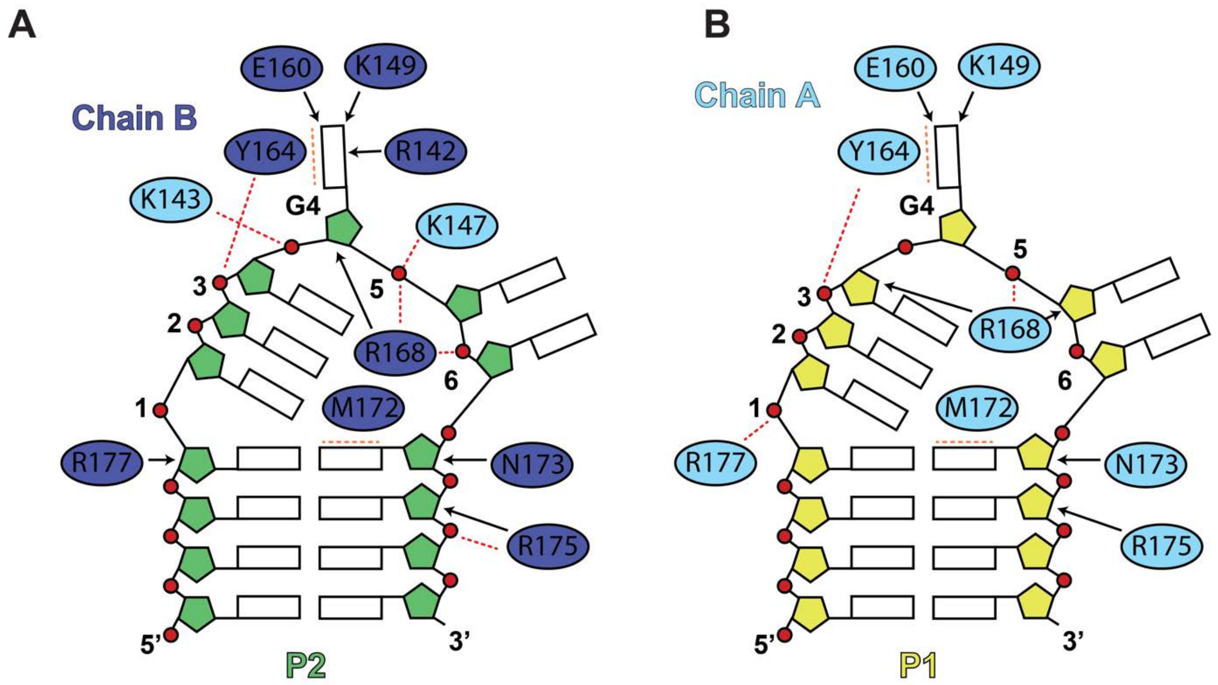

Supplementary Figure S10. Schematic showing the interactions between the EutP RNA hexaloops and EutV. (A) Residues from chain b (blue) contacting the P2 hexaloop (green) and (B) residues from chain a (cyan) contacting the P1 hexaloop (yellow). The only base specific interactions between the RNA and the EutV dimer is through the flipped G4 base. Hydrogen bond interactions are shown as black arrows, salt bridges as dashed red lines and π stacking / sulphur π interactions as dashed orange lines. Phosphate moieties are coloured red.

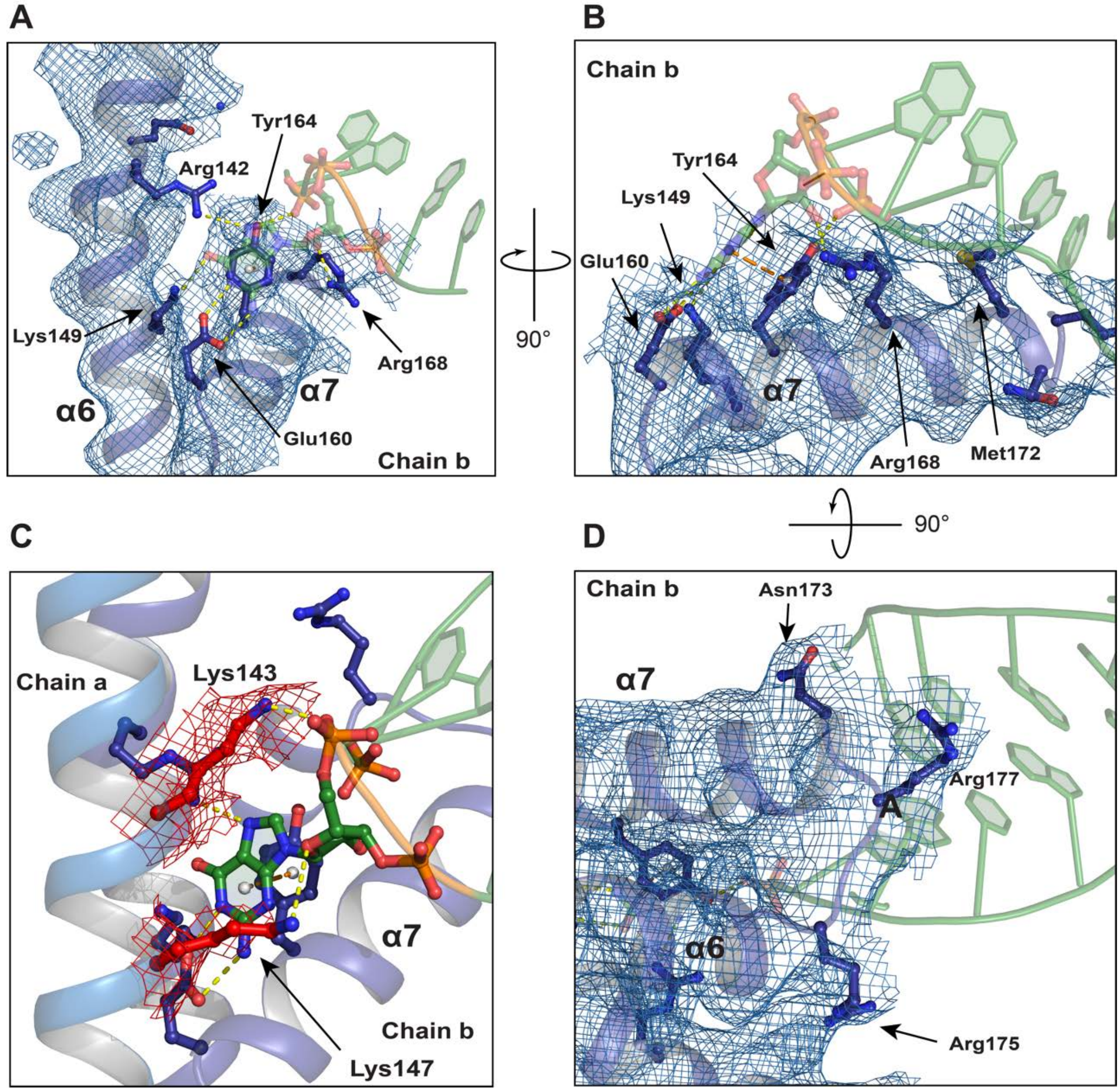

Supplementary Figure S11. Electron density of RNA binding residues from EutV. $2F_o$-F_c map contoured to 1 σ and coloured blue in all panels except (C) where coloured red. Hydrogen bonds shown as yellow dashes. π- π stacking represented as orange dashes.

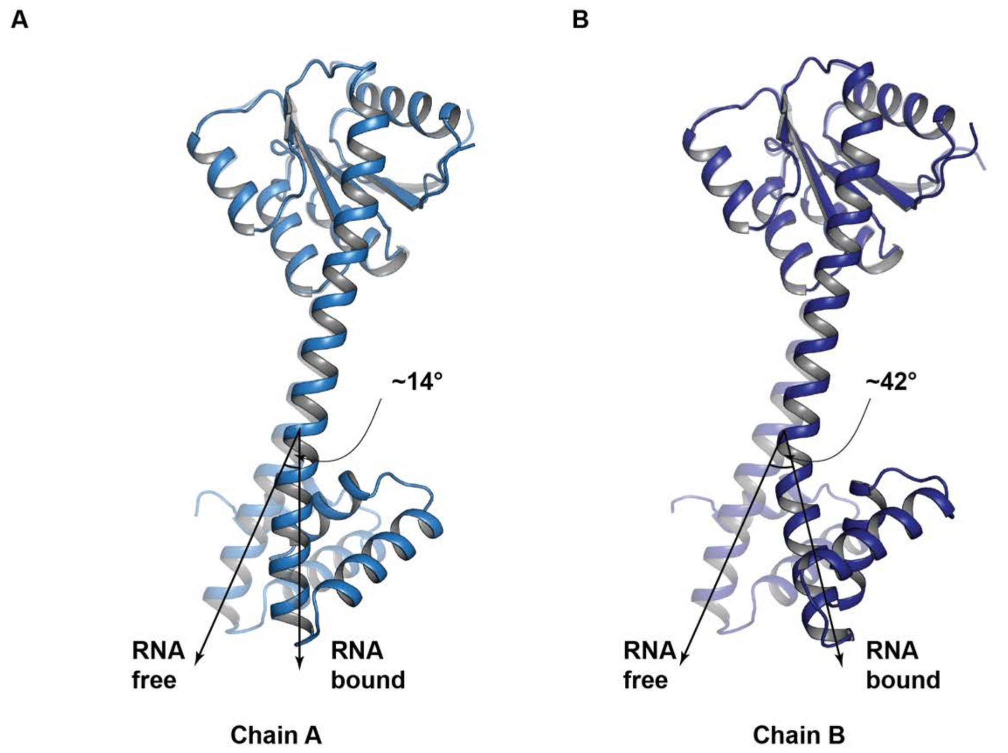

Supplementary Figure S12. Comparison of RNA free and bound EutV dimers. Comparison between each chain of the RNA free (transparent cartoon) and RNA bound (non-transparent cartoon) EutV dimers. (A) Chain A (sky-blue cartoon) moves approximately 14° upon RNA binding while (B) chain B (dark blue cartoon) moves over 40°.

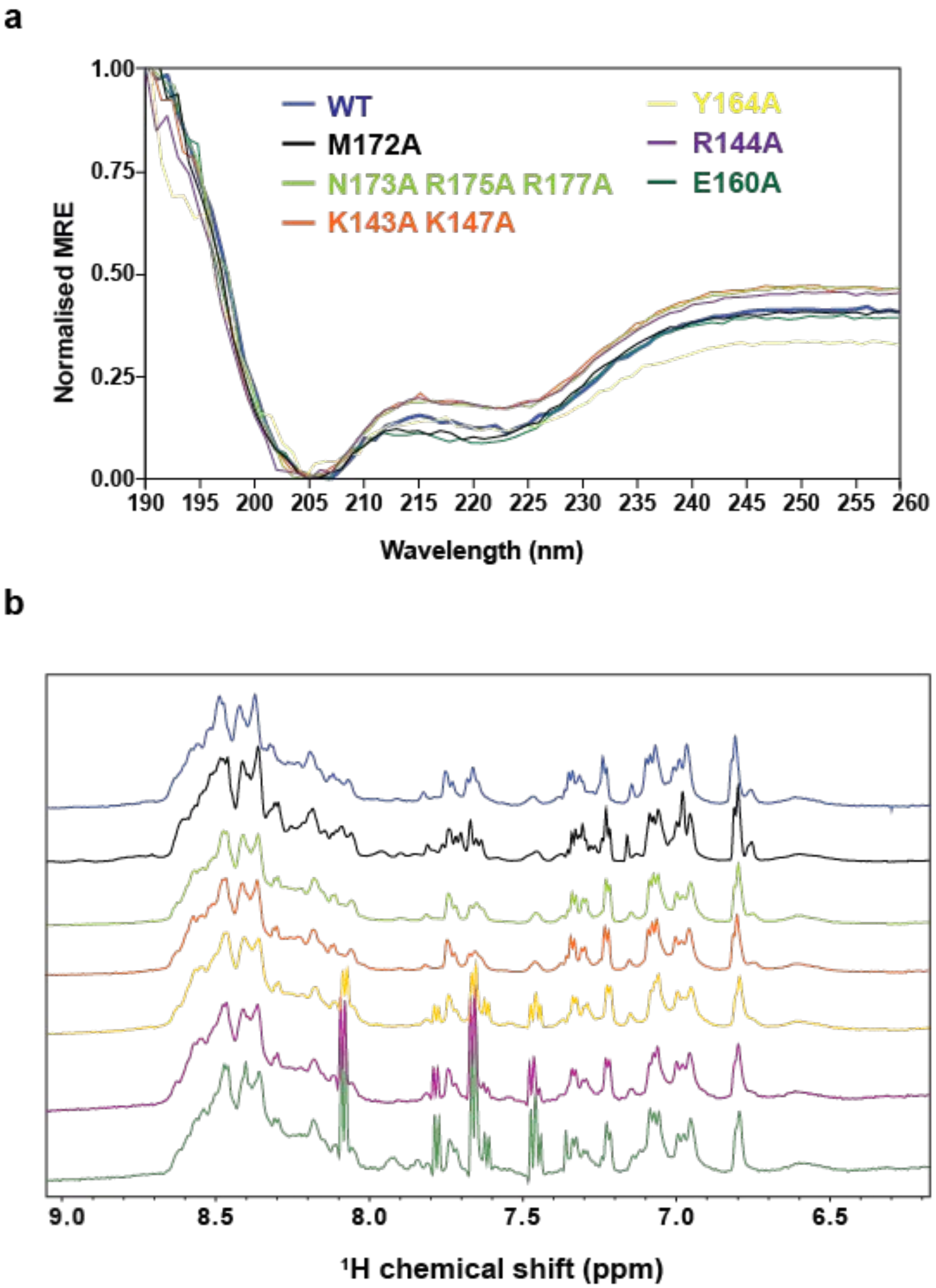

Supplementary Figure S13. UV CD and 1D 1H NMR spectrum of wild-type and mutant EutV constructs. Colouring is consistent across all panels. (A) Far-UV CD spectra normalised by setting the mean residue ellipticity (MRE) at 205 to 0. Spectra were recorded at 4°C with ~10 μM protein samples prepared in 10 mM HEPES pH 7, 300 mM NaF and 1 mM TCEP. Spectra are an average of three consecutive measurements. (B) 1D ^{1}H NMR spectra showing peaks in the amide (>6 ppm) region, recorded at 4° with 100 μM protein samples prepared in 50 mM HEPES pH 7, 300 mM NaCl, 1 mM TCEP

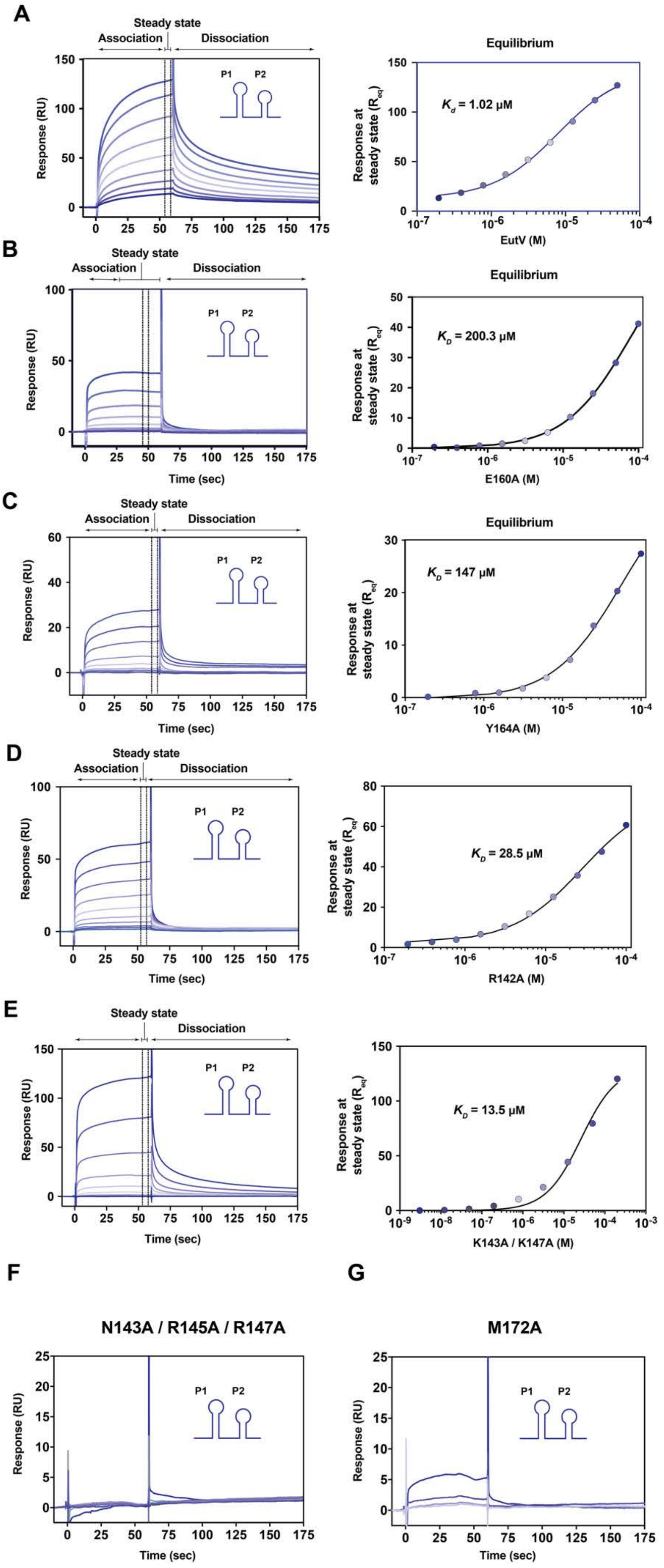
A
B
C
D
E
F
G
Steady state
Association
Dissociation
Response (RU)
Time (sec)
P1
P2
Equilibrium
Response at steady state (R_{eq})
K_d = 1.02 μM
EutV (M)
K_D = 200.3 μM
E160A (M)
K_D = 147 μM
Y164A (M)
K_D = 28.5 μM
R142A (M)
K_D = 13.5 μM
K143A / K147A (M)
N143A / R145A / R147A
M172A

Supplementary Figure S14. Representative SPR experiments of EutV WT and mutant constructs binding EutP RNA. ((A-E) Left panels) Representative normalised SPR sensorgrams of EutV WT and mutant proteins binding to EutP RNA. Association and dissociation regions are shown in the top most panel and apply for all sensorgrams. Sensorgrams show ten increasing EutV concentrations (0, 0.4, 0.8, 1.5, 3.125, 6.25, 12.5, 25, 50, 100 µM). (A-E, Right panels) Representative dose response plot of the interaction of EutV with immobilised RNA (as shown in corresponding left panel) at equilibrium fitted to a one-site Langmuir isotherm. The R_{max} parameter for the fragment binding was fixed during the curve fitting process and as estimated using the fitted maximum response for the positive control EutV WT. (F-G) Representative normalised SPR sensorgram of EutV N143A / R145A / R147 and EutV M172A respectively showing no binding.

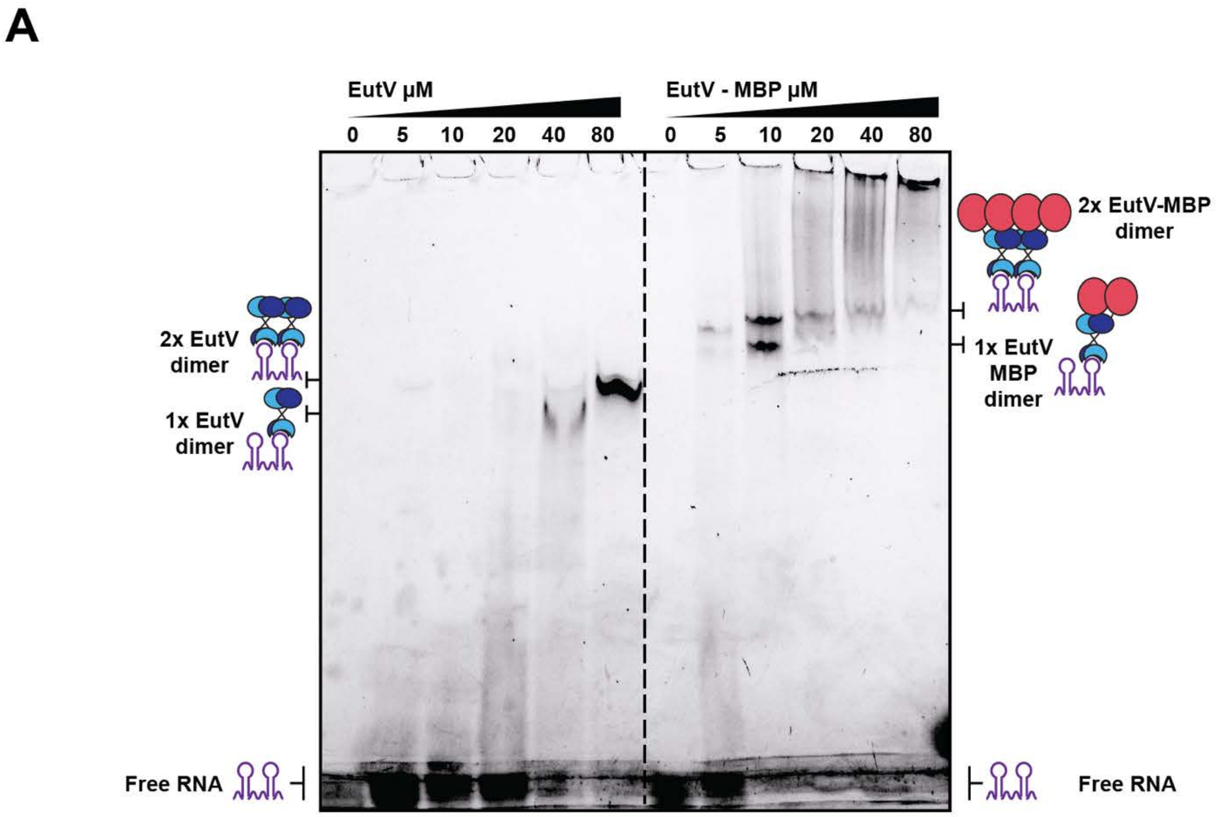

150 V 10 mA 60 mins

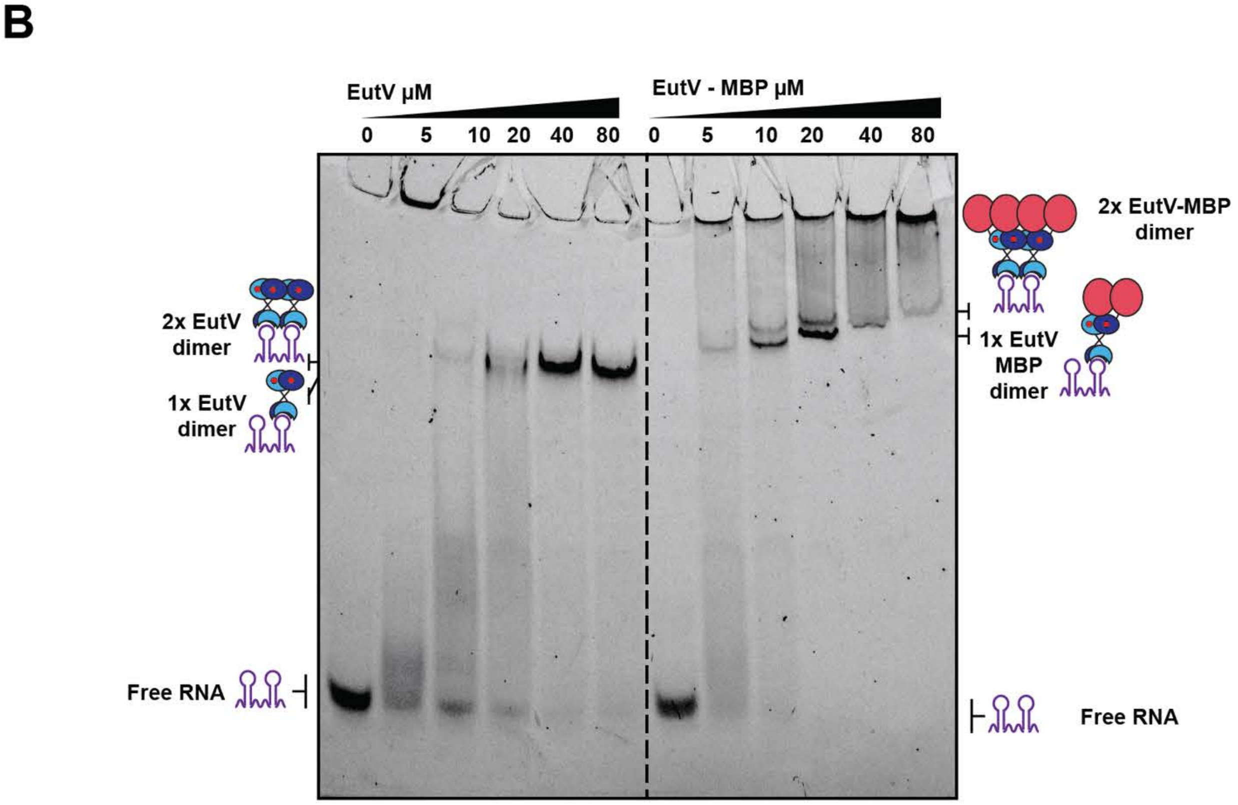

150 V 10 mA 45 mins

15 μM EutW + 5 mM ATP + 2.5 mM $MgCl_2$ + 1 mM EA

Supplementary Figure S15. Electromobility shift assay. (A) 25 nM of 5' Cy5 labelled RNA was incubated with increasing amounts of EutV or EutV MBP (0-80 µM) analysed on a 5% native polyacrylamide gel. Free RNA bands are indicated by the purple cartoon hairpin. Two distinct binding events were identified likely corresponding to a dimer binding at each hexaloop for both the EutV and EutV-MBP constructs. The first shift occurred between 5-20 µM and the second between 20-40 µM. (B) 25 nM of 5' Cy5 labelled RNA was incubated with increasing amounts of phosphorylated EutV or EutV-MBP (0-80 µM) and analysed on a 5% native polyacrylamide gel. To generate phosphorylated EutV, 80 µM EutV, 15 µM EutW, 5 mM ATP, 2.5 mM $MgCl_2$, 1 mM Ethanolamine (EA) were incubated prior to being analysed on a 5% native gel. Un-cleaved constructs indicated by pink MBP tag.

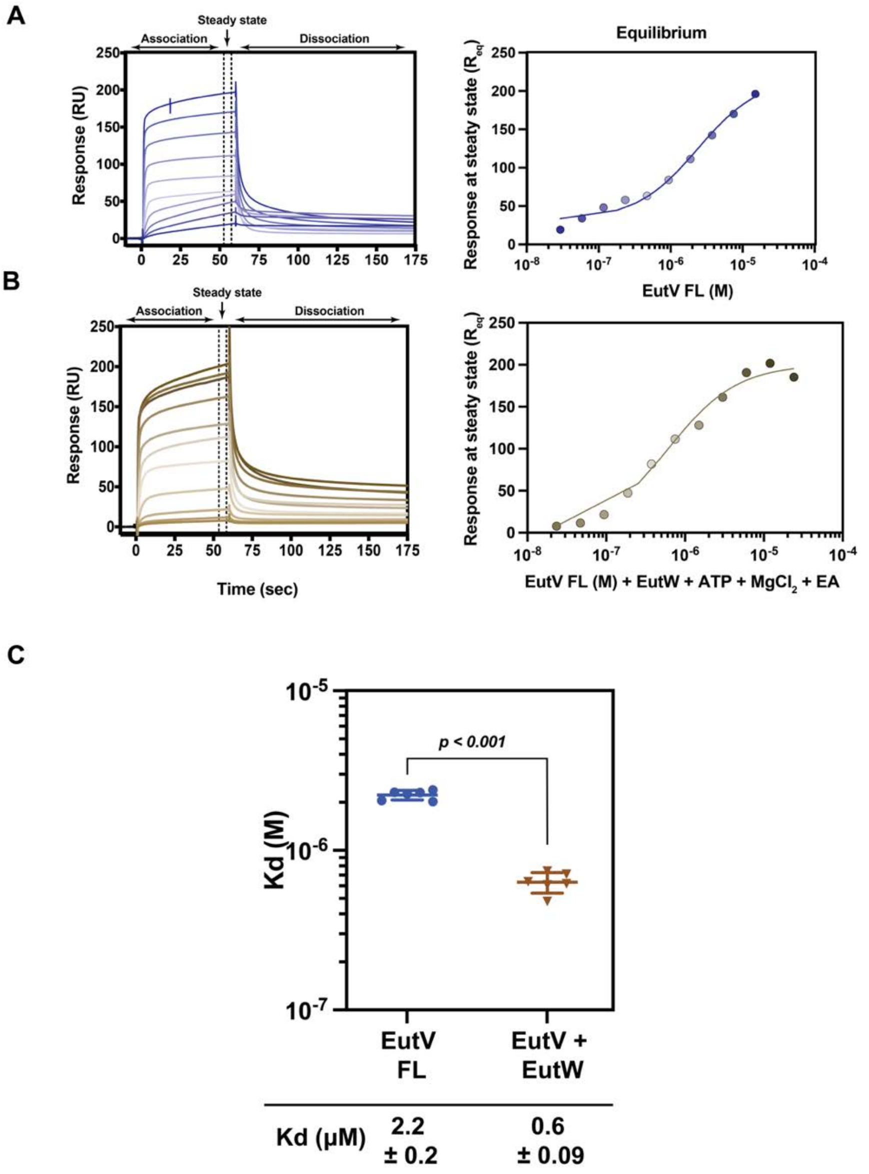

Supplementary Figure S16. ((A-B) Left panels) Representative SPR sensorgrams of EutV wildtype and phosphorylated EutV binding to EutP RNA. Association and dissociation regions are marked by black arrows. Sensorgrams show ten increasing concentrations (A-E, Right panels) Representative dose response plot of the interaction of EutV with immobilised RNA (as shown in corresponding left panel) at equilibrium fitted to a one-site Langmuir isotherm. (C) Average experimental K_D values from six SPR experiments are 2.2 ± 0.2 μM and 0.6 ±0.09 μM for EutP and EutV-P binding to EutP RNA respectively. The affinity of EutV and EutV-P to EutP RNA were compared with an independent sample *t*-test to maintain an over 5% error rate. The *p* value was < 0.001.

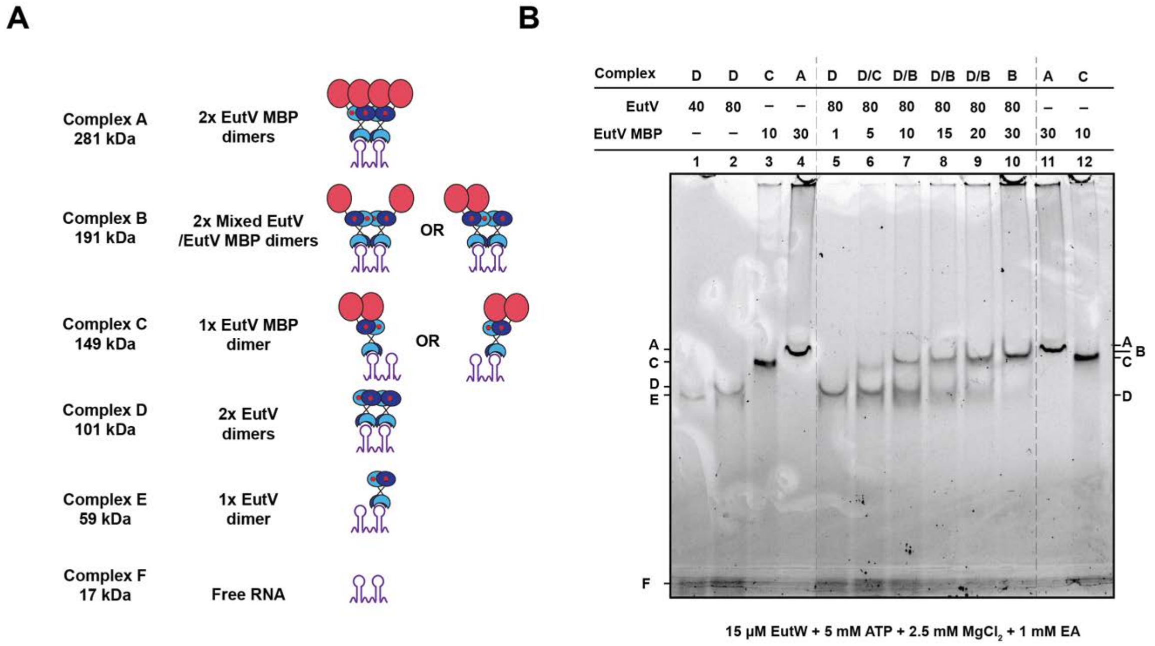

Supplementary Figure S17. Mixed EutV / EutV-MBP EMSA. (A) Schematic showing the expected binding scenarios and molecular weights for complexes in B. Un-cleaved constructs indicated by pink MBP tag. EutV dimer subunits shown in cyan and blue with phosphorylation represented by a red star. (B) Differing concentration of EutV and EutV-MBP were combined (as indicated) and phosphorylated by the addition of 15 µM EutW, 5 mM ATP, 2.5 mM $MgCl_2$, 1 mM Ethanolamine (EA). The mixtures were then incubated with 25 nM of 5’ Cy5 labelled RNA. Gels were electrophoresed for 80 minutes at 150 V and 20 mA. The predominant complex in each lane is indicated.

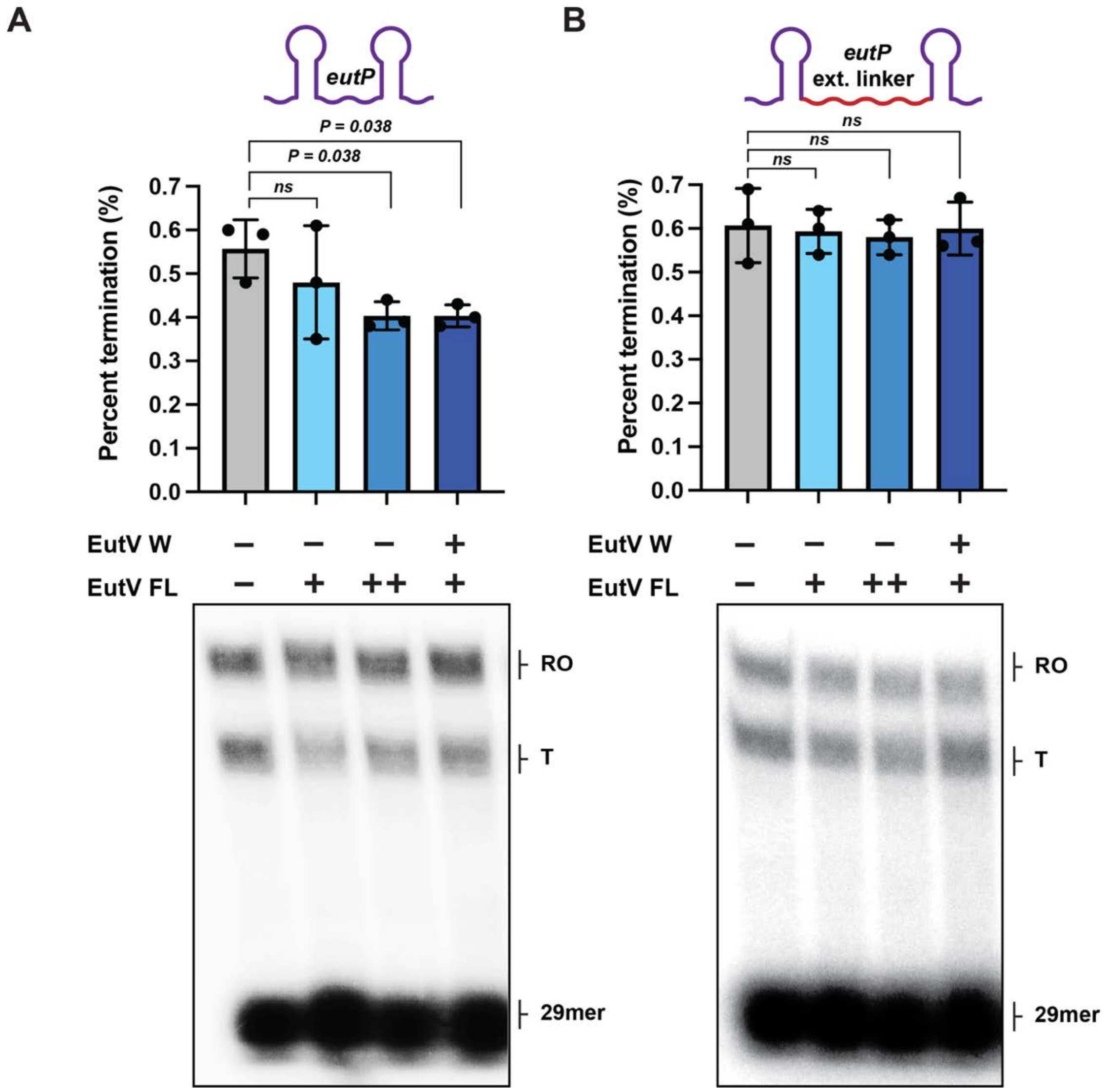

Supplementary Figure S18. *In vitro* antitermination assay. (A) Bar graph showing results from three independent *in vitro* transcription antitermination assays using the wild-type *eutP* template. Plus symbols represent the concentration of EutV used, either 1 μM or 10 μM. Transcription termination for each condition was compared using an analysis of variance (ANOVA), with the individual means being compared using a LSD test. Termination, in the absence of EutV (grey bar) is significantly different than termination in the presence of either 10 μM EutV (aqua bar) or 1 μM EutV with EutW (dark blue bar), $P < 0.05$. Representative gel of three independent replicates shown. (B). Bar graph showing results from three independent *in vitro* transcription antitermination assays using *eutP* extended template. Colouring, annotation, and statistical analysis as described in A. No significant difference was seen between conditions.

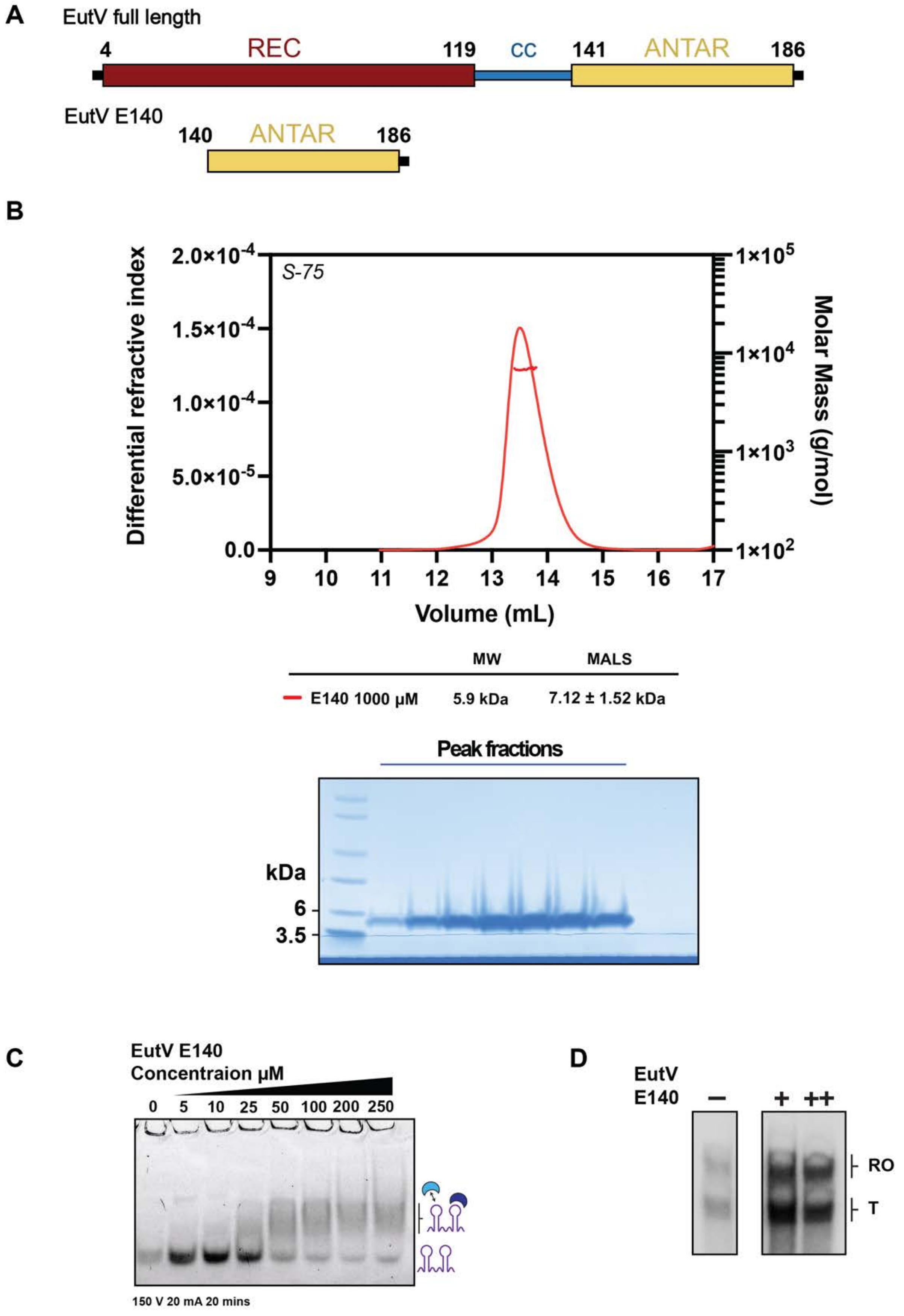

A
EutV full length
4
REC
119
cc
141
ANTAR
186
EutV E140
140
ANTAR
186
B
S-75
Differential refractive index
2.0×10^-4
1.5×10^-4
1.0×10^-4
5.0×10^-5
0.0
Molar Mass (g/mol)
1×10^5
1×10^4
1×10^3
1×10^2
9 10 11 12 13 14 15 16 17
Volume (mL)
MW
MALS
E140 1000 µM
5.9 kDa
7.12 ± 1.52 kDa
Peak fractions
kDa
6
3.5
C
EutV E140
Concentraion µM
0 5 10 25 50 100 200 250
150 V 20 mA 20 mins
D
EutV
E140
–
+
++
RO
T

Supplementary Figure S19. Monomeric EutV assays. (A) Schematic showing EutV E140 relative to the full-length construct (B) SEC-MALS analysis of EutV E140 showing normalised differential refractive index on the left y-axis and molecular mass estimates displayed as a scatter plots over each peak (right-hand y-axis). E140 was injected at 1000 μM (red trace) and separated on a Superdex75 10/300 Increase column. MALS analysis of the peaks reveals the protein is monomeric (7.12 ± 1.52 kDa). SDS-PAGE showing elution to be highly pure. (C) Electromobility shift assay. 25 nM of 5' Cy5 labelled RNA was incubated with increasing amounts of EutV E140 (0-250 μM) and analysed on a 5% native polyacrylamide gel. Free RNA bands are indicated by the purple cartoon hairpin. Weak binding was between 25 μM and 50 μM however saturation could not be achieved when in excess of 250 μM was used, highlighting the importance of dimerisation in RNA binding (D) *In vitro* antitermination assay using the wild-type *eutP* template and E140. Plus symbols represent the concentration of E140 used, either 4 μM or 40 μM. No antitermination of transcription was seen.

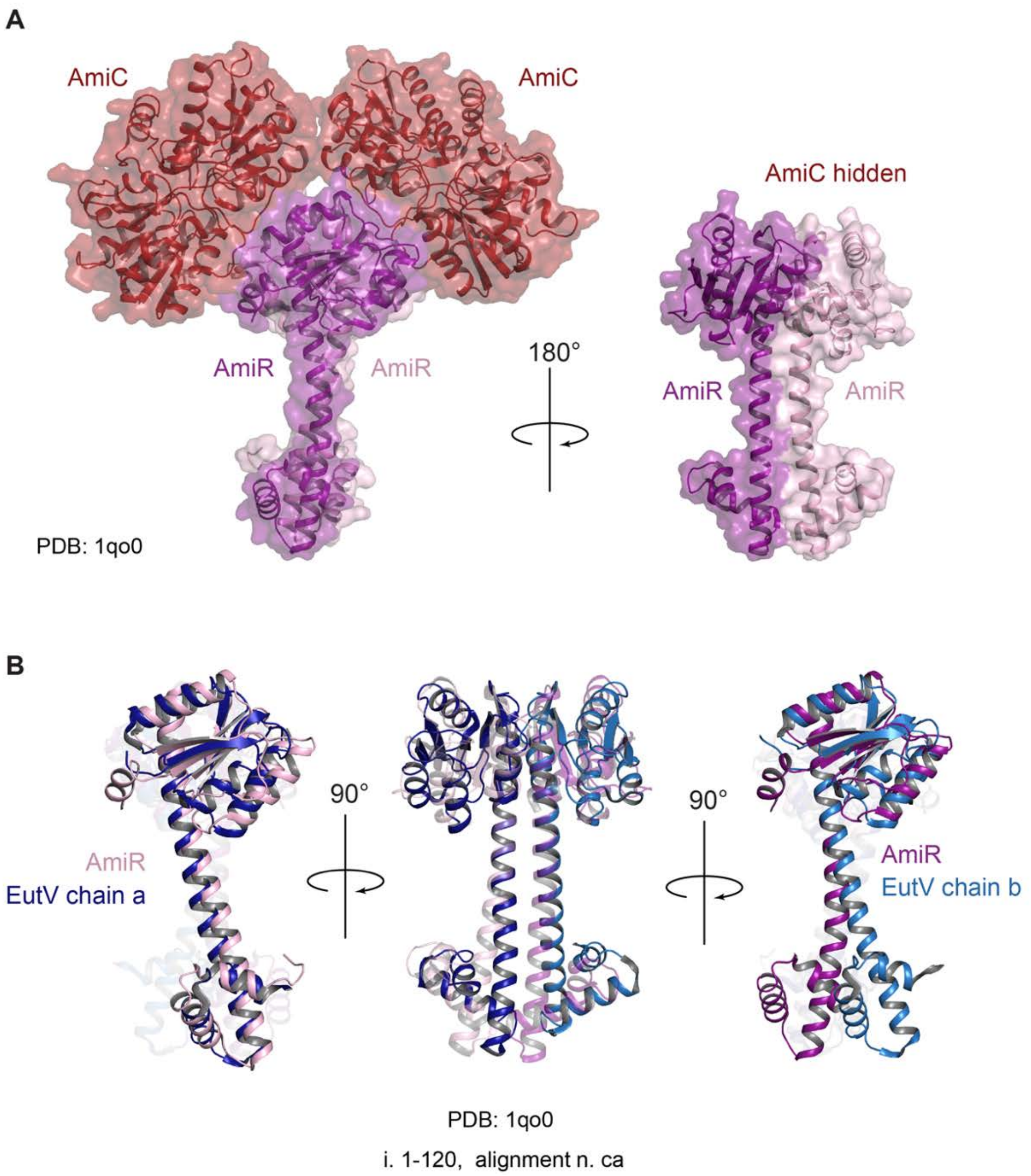

Supplementary Figure S20. Structural alignment of EutV and AmiR. (A) Crystal structure of AmiC/AmiR complex (pdb: 1qo0) with AmiC coloured in red and AmiR in a dark and light shades of pink. (B) Structural alignment of EutV and AmiR showing the asymmetric nature of the AmiR (attributed to crystal packing limitations (4)) dimer compared to symmetric EutV dimer. EutV is coloured in a light and dark shades of blue.

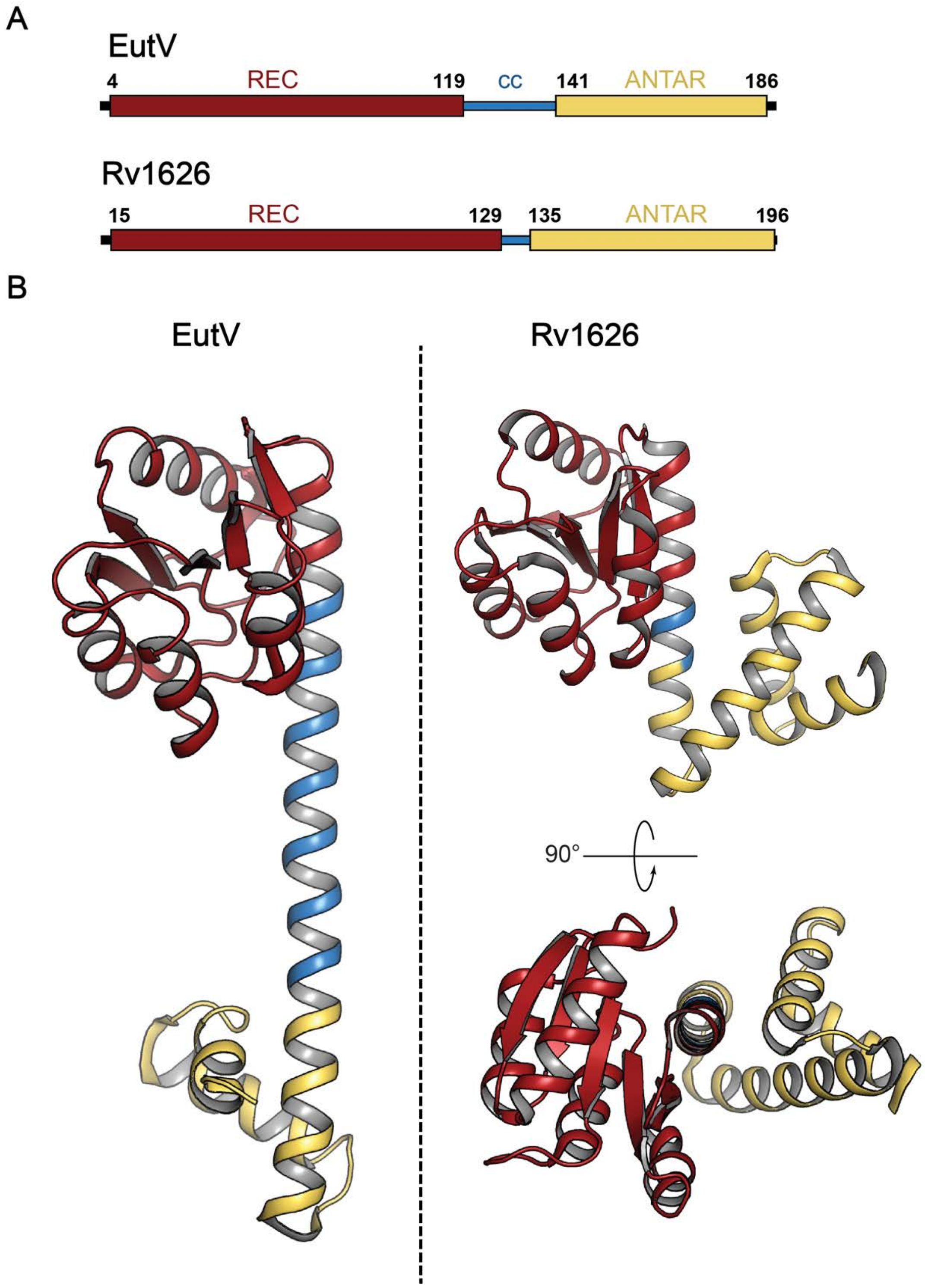

Supplementary Figure S21. Structural comparison of EutV and Rv1626. (A) Domain organisation of EutV and the putative transcription anti-terminator Rv1626 from *M. tuberculosis* (5) (B) Comparison of a single chain of the RNA free EutV dimer and Rv1626. Both proteins are coloured the same way with the receiver (REC) domain is shown in red, the coiled-coil domain in blue and the ANTAR domain in yellow.

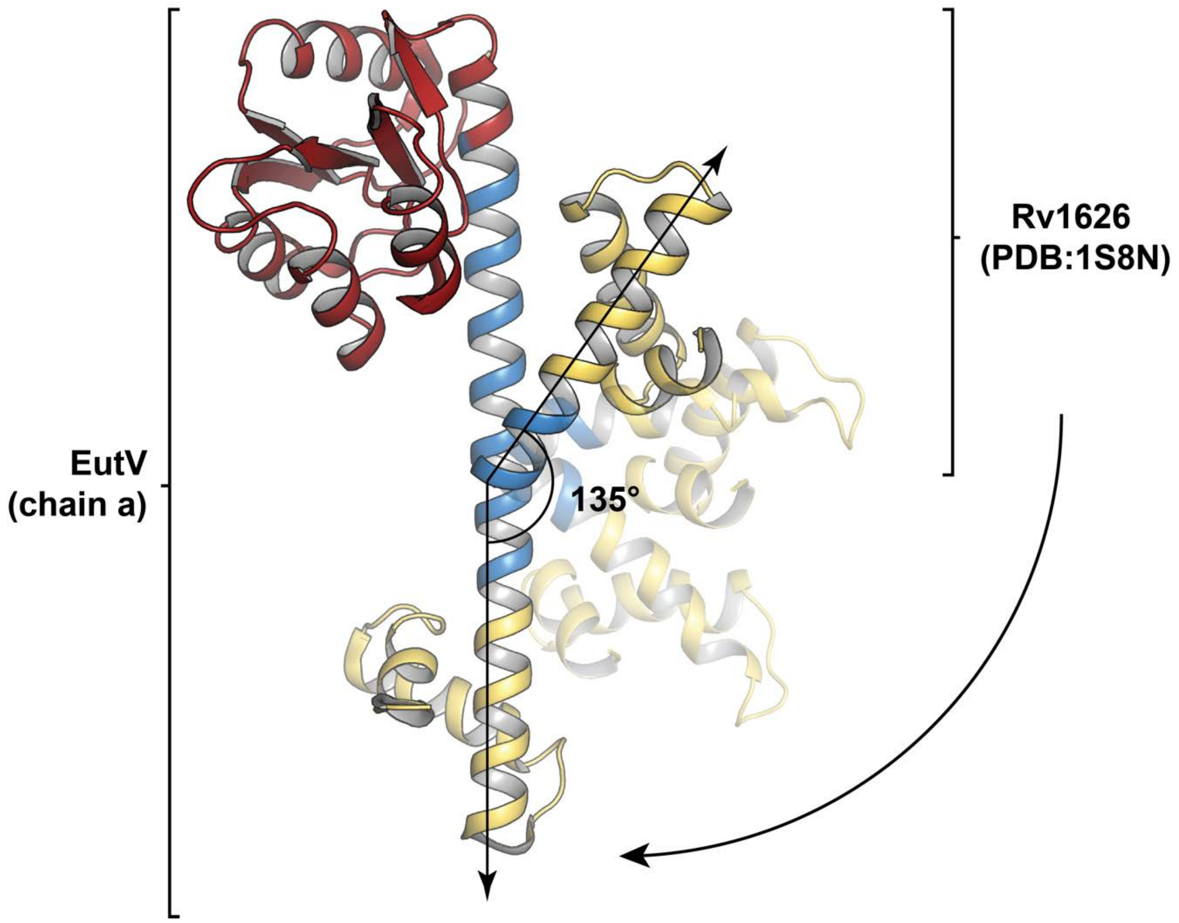

Supplementary Figure S22**.** Modelled extension of the α5- α6 helix required to transition EutV from a monomer to a dimer. Rv1626 (pdb: 1s8n) was used to model the EutV monomer seen in solution.

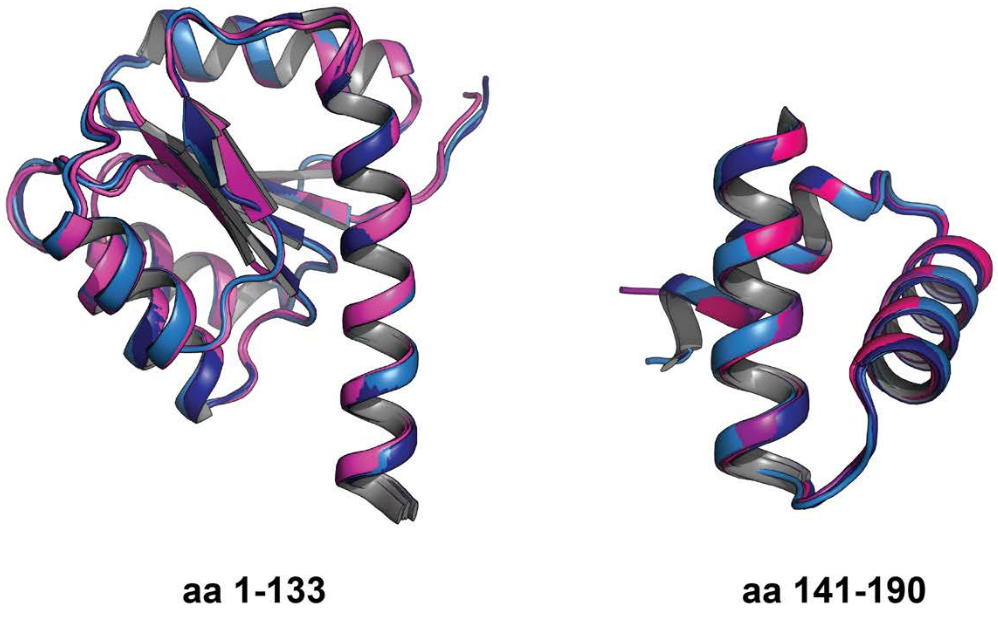

Supplementary Figure S23. Comparison of REC domains and ANTAR domains of EutV free and the EutV:EutP RNA structure. Chain A and B of EutV:EutP RNA complex show no significant difference when compared to both chains of the RNA-free structure (RMSD Cα atoms 0.36 Å and 0.30 Å) for REC and ANTAR domains respectively. EutV RNA free is coloured in a light and dark shades of pink for chain A and B respectively while EutV:EutP RNA is coloured in a light and dark shades of blue for chain A and B respectively.

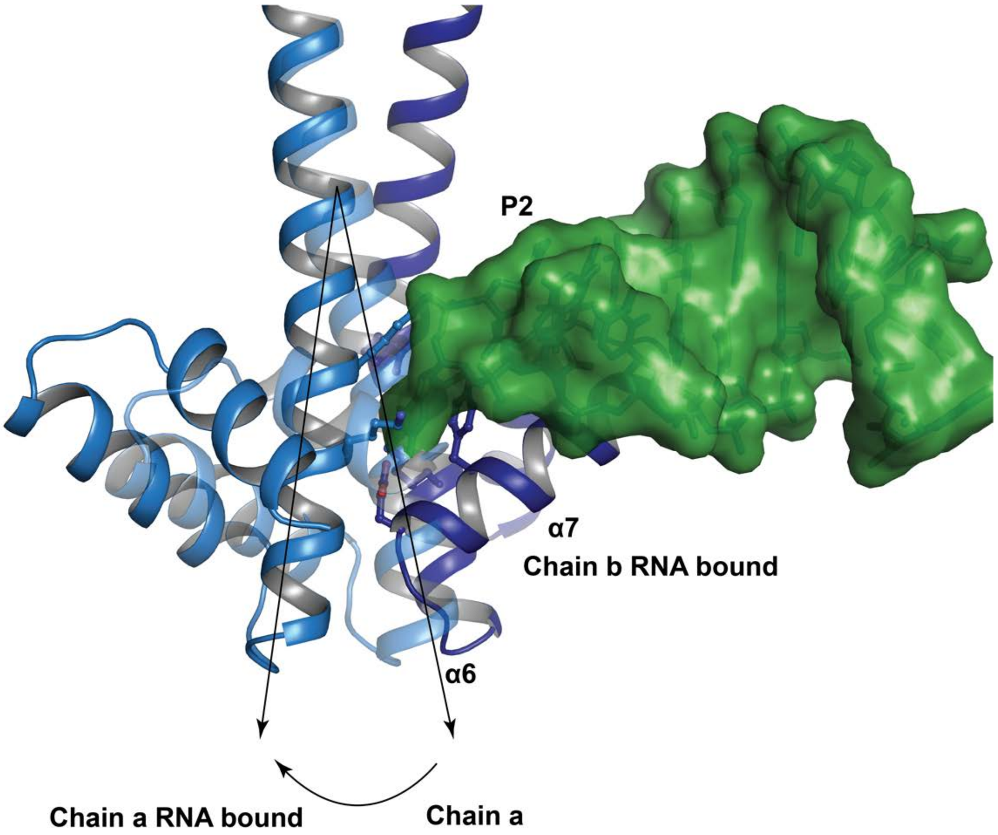

Supplementary Figure S24. Helical flexing upon RNA binding. Cartoon showing flexing of α6 between RNA free (transparent) and RNA bound EutV structures is required to expose the RNA binding surface of α7.

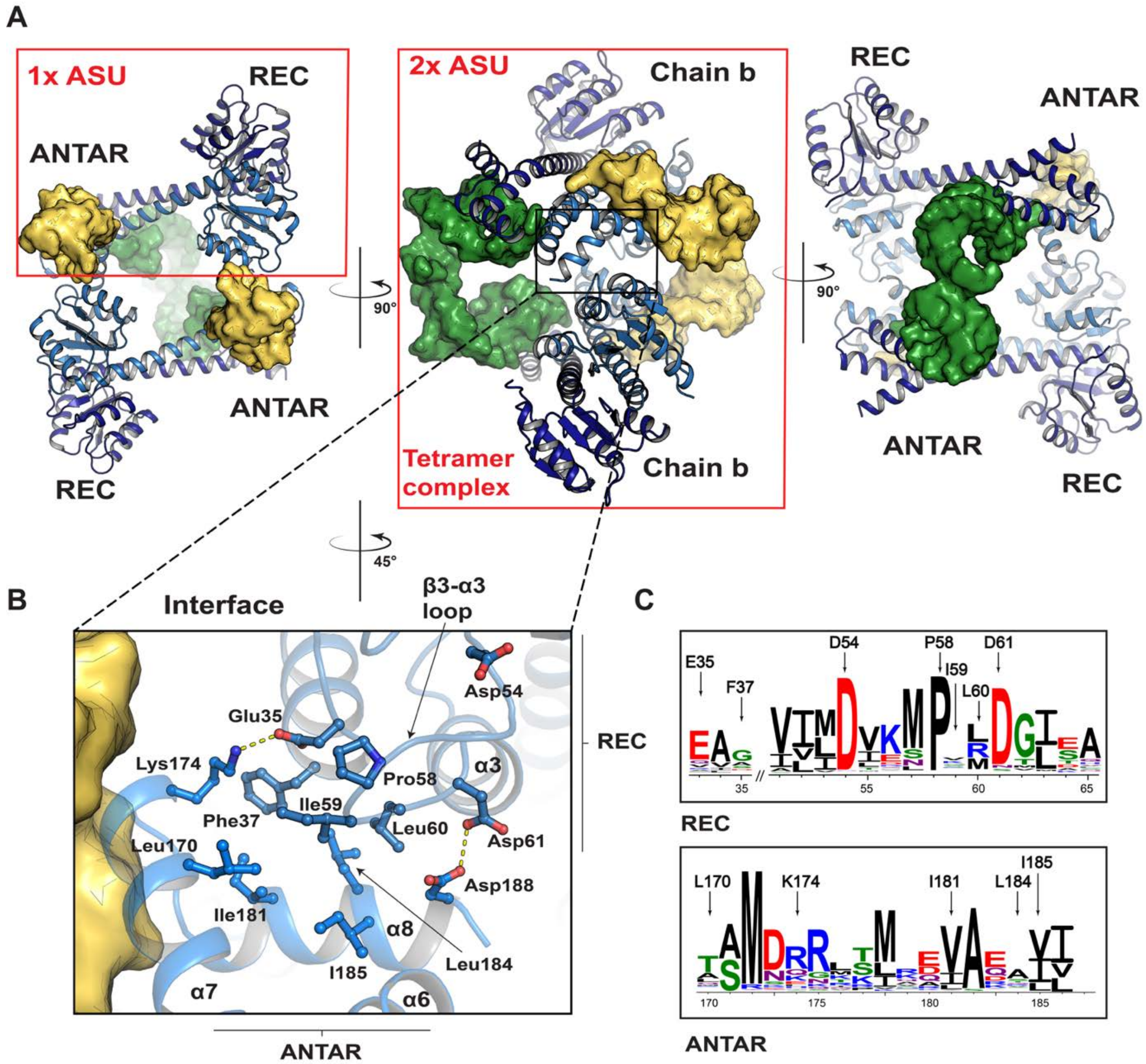

Supplementary Figure S25. Interaction of potential EutV tetramer within crystal lattice. (A) Lattice packing arrangement of EutV tetramer complex. Two EutV dimers pack head to tail within the lattice. Symmetry related RNA shown as yellow and green surface representation. EutV chain a and b shown as sky-blue and dark blue pymol cartoon respectively. (B) Crystal contact between chain a of two symmetry related dimers (within possible tetramer complex). Hydrogen bonds represented as yellow dashes. (C)) Multiple sequence alignment 2332 ANTAR domain proteins containing a N-terminal REC domain and C-terminal ANTAR domain shown as a WebLogo (6).

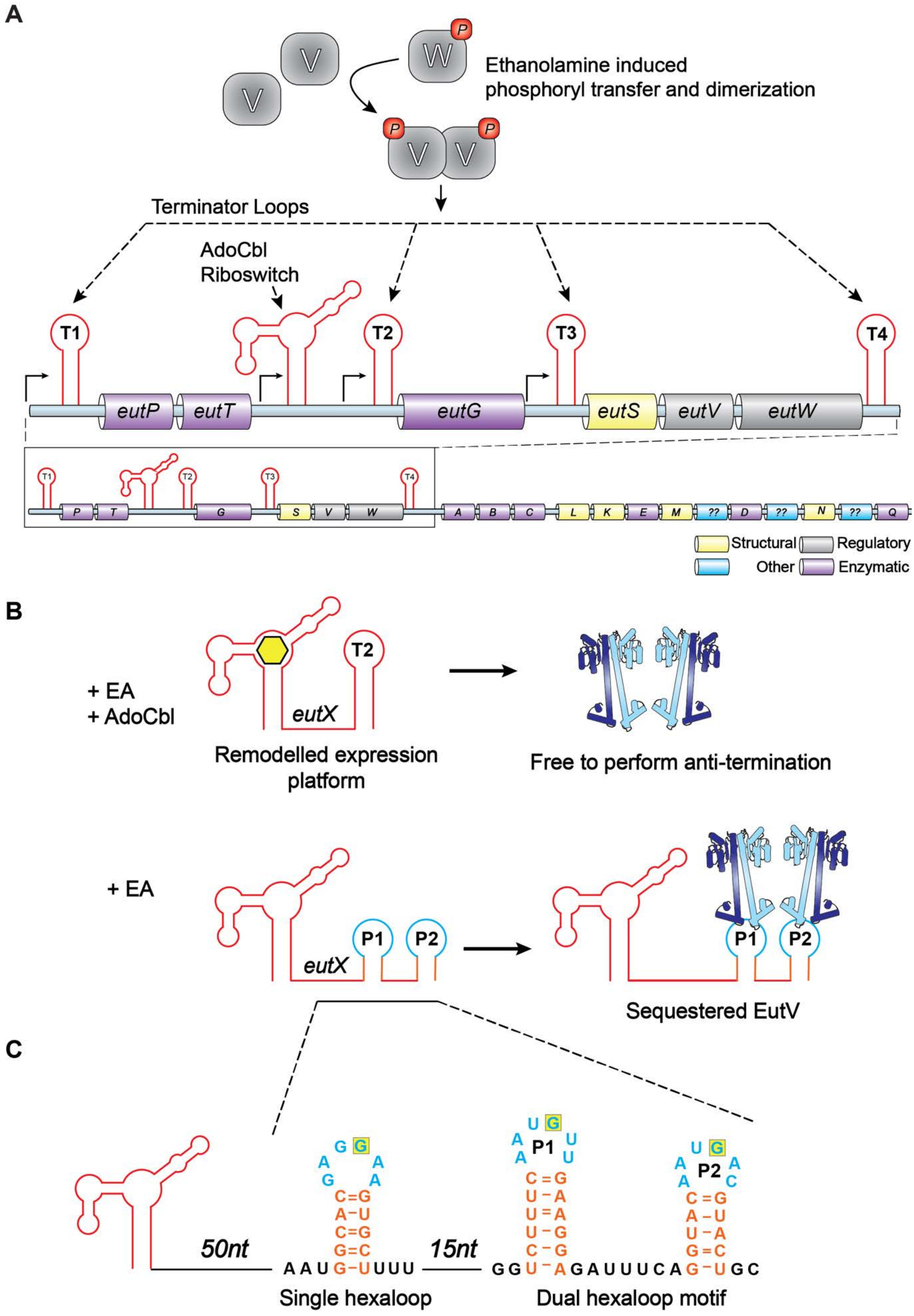
A
V
V
W
P
Ethanolamine induced
phosphoryl transfer and dimerization
Terminator Loops
AdoCbl
Riboswitch
T1
T2
T3
T4
eutP
eutT
eutG
eutS
eutV
eutW
P
T
G
S
V
W
A
B
C
L
K
E
M
??
D
N
Q
Structural
Regulatory
Other
Enzymatic
B
+ EA
+ AdoCbl
eutX
Remodelled expression platform
Free to perform anti-termination
+ EA
P1
P2
Sequestered EutV
C
50nt
15nt
AAUG-UUUU
GGU-AGAUUUCAG-UGC
Single hexaloop
Dual hexaloop motif

Supplementary Figure S26. EutX sRNA regulation. (A) Gene organisation of the *eut* operon highlighting the *eutT-eutG* intragenic region (B) Model for *eutX* transcript regulation of the *eut* operon by sequestration of dimeric EutV as proposed by Deboy et al. (7) Adenosylcobalamin (AdoCbl)(yellow hexagon), and essential cofactor for ethanolamine catabolism, binds the AdoCbl riboswitch (red cartoon) remodelling the expression platform to promote T-loop formation (single red loop). When ethanolamine is present, but AdoCbl absent, dimeric EutV is sequested by a *eutX* transcript containing hexaloops. (C) Cartoon showing additional single hexaloop structure identified in the 131nt region between AdoCbl riboswitch and dual hairpin motif. Hairpins and hexaloops shown in orange and blue respectively. Position 4 of the hexaloop coloured in yellow. Sequence analysed using MFOLD (8).

Supplementary Movie 1: Shows the RNA binding residues, flexing of helices and the interactions with RNA between the EutV dimer crystal structure, the transition state model and RNA bound crystal structure. Morphs between states were generated in PyMOL Transitions state model is composed of chain a of the RNA free structure with chain a of the RNA bound structure.

Supplementary Table 1: X-ray diffraction and crystal structure refinement statistics for EutV alone and RNA bound complex

	**EutV**	**EutV:RNA bound**
PDB entry	6WSH	6WW6
**Data collection**		
Space group	$P2_1\ 2_1 2_1$	$I4_1\ 3\ 2$
Cell dimensions		
a, *b*, *c* (Å)	54.54, 85.69, 97.49	258.48, 258.48, 258.48
α, *β*, *γ* (°)	90, 90, 90	90, 90, 90
Resolution (Å)[a]	42.85–2.12	47.19–3.80
	(2.18–2.12)[a]	(4.25–3.80)[a]
R_{merge}	0.195 (0.568)	0.151 (0.866)
$CC_{1/2}$	0.984 (0.377)	0.998 (0.733)
I/σ_I	6.0 (2.09)	9.1 (2.3)
Completeness (%)	100 (100)	99.5 (98.9)
Redundancy	4.8 (4.9)	6.7 (6.7)
**Refinement**		
Resolution (Å)	42.85–2.12	47.19–3.80
No. of reflections	42 169	98 478
R_{work}	0.2392 (0.3152)	0.2331 (0.3215)
R_{free}	0.2405 (0.3165)	0.2414 (0.3484)
No. of atoms		
Protein	6092	4481
Water	360	0
B-factors (Å^2)		
Protein	22.62	158

RNA	-	214
Water	28.06	-
RMSD[b]		
Bond lengths (Å)	0.008	0.006
Bond angles (°)	1.24	0.958
Ramachandran		
Favored (%)	95.74	96.30
Allowed (%)	4.26	3.17
Disallowed (%)	0.00	0.53
Validation		
MolProbity score	0.92	1.33
Clashscore	1.47	4.85
Poor rotamers (%)	0.00	0.00

[a]Statistics for the highest resolution shell are shown in parentheses.
[b]Categories were defined using PHENIX.

Supplementary References

1. Ramesh, A., DebRoy, S., Goodson, J.R., Fox, K.A., Faz, H., Garsin, D.A. and Winkler, W.C. (2012) The mechanism for RNA recognition by ANTAR regulators of gene expression. *PLoS Genet.*, **8**, e1002666-e1002666.
2. Popenda, M., Szachniuk, M., Antczak, M., Purzycka, K.J., Lukasiak, P., Bartol, N., Blazewicz, J. and Adamiak, R.W. (2012) Automated 3D structure composition for large RNAs. *Nucleic Acids Res.*, **40**, e112-e112.
3. Afonine, P.V., Grosse-Kunstleve, R.W., Echols, N., Headd, J.J., Moriarty, N.W., Mustyakimov, M., Terwilliger, T.C., Urzhumtsev, A., Zwart, P.H. and Adams, P.D. (2012) Towards automated crystallographic structure refinement with phenix. refine. *Acta Cryst. D*, **68**, 352-367.
4. O'Hara, B.P., Norman, R.A., Wan, P.T., Roe, S.M., Barrett, T.E., Drew, R.E. and Pearl, L.H. (1999) Crystal structure and induction mechanism of AmiC–AmiR: a ligand-regulated transcription antitermination complex. *EMBO J.*, **18**, 5175-5186.
5. Morth, J.P., Gosmann, S., Nowak, E. and Tucker, P.A. (2005) A novel two-component system found in Mycobacterium tuberculosis. *FEBS Lett.*, **579**, 4145-4148.
6. Crooks, G.E., Hon, G., Chandonia, J.-M. and Brenner, S.E. (2004) WebLogo: a sequence logo generator. *Genome Res.*, **14**, 1188-1190.
7. DebRoy, S., Gebbie, M., Ramesh, A., Goodson, J.R., Cruz, M.R., van Hoof, A., Winkler, W.C. and Garsin, D.A. (2014) A riboswitch-containing sRNA controls gene expression by sequestration of a response regulator. *Science*, **345**, 937-940.
8. Zuker, M. (2003) Mfold web server for nucleic acid folding and hybridization prediction. *Nucleic Acids Res.*, **31**, 3406-3415.
